# Application and progress of 3D printed biomaterials in osteoporosis

**DOI:** 10.3389/fbioe.2025.1541746

**Published:** 2025-02-04

**Authors:** Chenxu Wang, Aiguo Liu, Ziwen Zhao, Ting Ying, Shuang Deng, Zhen Jian, Xu Zhang, Chengqing Yi, Dejian Li

**Affiliations:** ^1^ Department of Orthopedics, Shanghai Pudong Hospital, Fudan University Pudong Medical Center, Shanghai, China; ^2^ Department of Orthopedics, The First Affiliated Hospital of Henan University, Kaifeng, China

**Keywords:** 3D printing, biomaterials, osteoporosis, reactive oxygen species, bone

## Abstract

Osteoporosis results from a disruption in skeletal homeostasis caused by an imbalance between bone resorption and bone formation. Conventional treatments, such as pharmaceutical drugs and hormone replacement therapy, often yield suboptimal results and are frequently associated with side effects. Recently, biomaterial-based approaches have gained attention as promising alternatives for managing osteoporosis. This review summarizes the current advancements in 3D-printed biomaterials designed for osteoporosis treatment. The benefits of biomaterial-based approaches compared to traditional systemic drug therapies are discussed. These 3D-printed materials can be broadly categorized based on their functionalities, including promoting osteogenesis, reducing inflammation, exhibiting antioxidant properties, and inhibiting osteoclast activity. 3D printing has the advantages of speed, precision, personalization, etc. It is able to satisfy the requirements of irregular geometry, differentiated composition, and multilayered structure of articular osteochondral scaffolds with boundary layer structure. The limitations of existing biomaterials are critically analyzed and future directions for biomaterial-based therapies are considered.

## 1 Introduction

Osteoporosis is a systemic bone metabolic disorder marked by chronic trabecular bone loss and heightened fracture risk. It is defined by reduced bone mass and the progressive deterioration of bone tissue microarchitecture. The common sites for osteoporotic fractures are the spine (vertebrae), hip (proximal femur) or wrist (distal forearm). As the global population ages, osteoporosis has emerged as a significant medical and social challenge, particularly affecting the elderly and postmenopausal individuals with increased prevalence (Codrea et al., 2021). Each year, nearly 200 million patients are diagnosed with osteoporosis worldwide, and approximately 9 million osteoporotic fractures occur (Xie et al., 2019), with an increasing number of older adults susceptible to fragility fractures Conventional treatments for osteoporosis are usually limited to pharmacologic therapy with anti-resorptive and anabolic drugs. Such as calcitonin and bisphosphonates (alendronate) are successful in increasing bone mass and limiting fracture risk. However, the efficacy of pharmacologic treatments is limited by side effects associated with long-term drug therapy and decreased patient compliance with medication (Vargas-Franco et al., 2018).

In recent years, advancements in medical imaging, digital information technology, and manufacturing techniques have driven increasing interest in the application of 3D printing for the treatment of various diseases. 3D printing, also known as additive manufacturing, is a technology that fabricates materials—whether metallic, non-metallic, or biomaterials—by layering them according to a digital model derived from the patient’s anatomy. This approach enables the creation of highly customized, three-dimensional structures tailored to individual patient needs, offering significant potential in personalized medicine and regenerative treatments (Lei et al., 2023). 3D printing technology offers exceptional flexibility, characterized by short operation times, high efficiency, and ease of use. It facilitates the precise delivery of therapeutic agents directly to osteoporotic bone defect sites, significantly contributing to improved osseointegration and accelerated healing. 3D printing technology can be used to provide a healing factor to the bone, which is essential to optimize the rate of osseointegration/healing (Koons et al., 2020/08).

**SCHEME 1:**
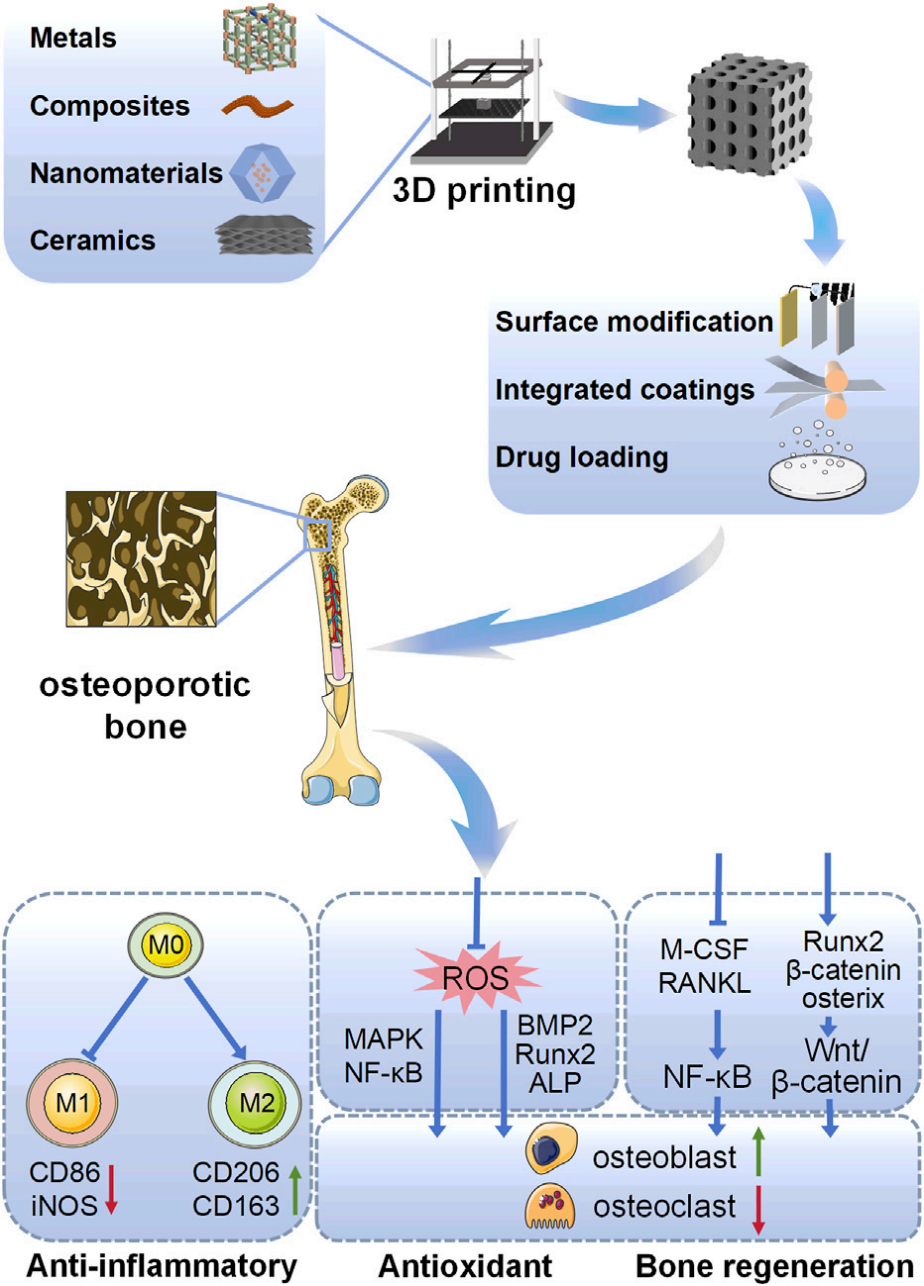
Schematic representation of different types of 3D printed biomaterials targeting the promotion of osteoporotic osseointegration in this review, including different material types, surface modifications, and mechanisms of action.

The utilization of 3D printing in addressing bone tissue disorders offers numerous advantages, including precise spatial control of cell and material placement, the capacity to design complex tissue-interfacing surfaces, customization of mechanical and biological characteristics, and the seamless integration of scaffold degradation rates with structural optimization (Feng et al., 2021). Furthermore, advancements in 3D printing technology, supported by computer-aided design (CAD) modeling, have significantly enhanced the precision and reproducibility of scaffold fabrication, enabling meticulous control of porosity at both micro- and macro-level scales. While macroporosity is important for rapid cellular infiltration, vascularized oxygen and nutrient exchange, microporosity is critical for designing release profiles (Petre et al., 2021). This review outlines the physiology, pathology, and repair mechanisms of osteoporosis, while providing a comprehensive overview of advancements in 3D-printed materials for its treatment (Scheme 1).

## 2 Pathogenesis of osteoporosis

### 2.1 Bone remodeling

Osteoporosis is primarily caused by a disruption in the balance between osteogenesis (bone formation) and osteoclastogenesis (bone resorption). Bone is composed of an outer dense cortical layer and an inner porous cancellous layer, both of which contribute to its overall strength. Bone tissue is composed of various cellular and extracellular components, including osteoblasts, osteoclasts, stem cells, and a bone matrix. The matrix primarily consists of calcium, phosphorus, inorganic salts, and collagen, providing the structural and functional foundation of bone. Osteoclasts are responsible for bone resorption, breaking down bone tissue to release minerals into the bloodstream. At the same time osteoblasts facilitate new bone formation, maintaining the balance of bone remodeling and ensuring skeletal integrity (Kenkre and Bassett, 2018). Osteoblasts and osteoclasts work in concert to maintain bone health and structural integrity through a dynamic process known as bone remodeling or bone turnover. This process ensures the continuous renewal of bone tissue by balancing bone resorption by osteoclasts with bone formation by osteoblasts. Disruption in the activity of these cells can result in microstructural degradation, diminished bone strength, and an elevated risk of fragility fractures, highlighting the importance of their coordinated function in skeletal health (Liang et al., 2022).

**FIGURE 1:**
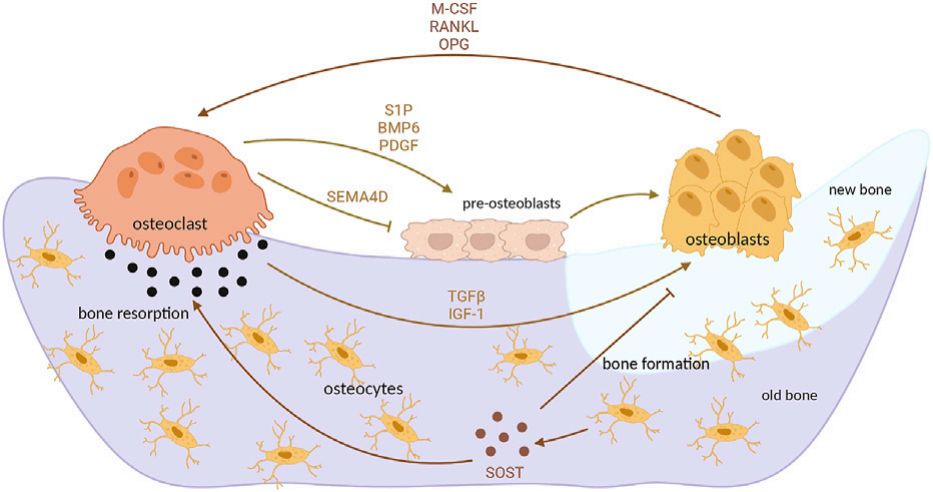
Interaction between osteoblasts and osteoclasts during bone remodeling (Bordukalo-Nikšić et al., 2022). During bone remodeling, osteoclasts play a key role in initiating the process by releasing signaling molecules such as TGF-β and IGF-1. These factors enhance osteoblast activity and promote subsequent bone formation. Additionally, osteoclasts secrete sphingosine-1-phosphate (S1P) and platelet-derived growth factor (PDGF), which stimulate osteoblast differentiation. In contrast, they also produce semaphorin 4D (SEMA4D), which inhibits osteoblast activity, highlighting their dual regulatory role. Osteoblasts, in turn, secrete cytokines such as macrophage colony-stimulating factor (M-CSF), receptor activator of nuclear factor kappa-B ligand (RANKL), and osteoprotegerin (OPG). These molecules regulate osteoclast differentiation and activity by maintaining a balance between bone resorption and formation. Osteogenic precursor cells differentiate into mature osteoblasts, and some osteoblasts further transform into osteocytes embedded within the bone matrix. Osteocytes secrete sclerostin (SOST), which promotes osteoclastogenesis while inhibiting further osteoblast differentiation, providing a negative feedback mechanism in bone remodeling. This dynamic interplay between osteogenesis and osteoclastogenesis ensures a balance critical for maintaining the long-term structural stability of bone tissue, allowing it to adapt to mechanical demands while preserving its integrity. (Copyright ^©^ 2022 Bordukalo-Nikšić, Kufner and Vukičević).

Physiological bone remodeling occurs in five distinct phases (Lei et al., 2023): 1) During the activation phase, osteoblasts play a pivotal role in sensing and transmitting mechanical signals. These signals, along with local mechanical stimuli or hormonal cues, drive osteoclast differentiation. This process is accompanied by a marked increase in systemic and local bioregulators that facilitate osteoclast formation. Key regulators include parathyroid hormone (PTH), receptor activator of nuclear factor-κB ligand (RANKL), and macrophage colony-stimulating factor (M-CSF), all of which orchestrate the recruitment and activation of osteoclast precursors. 2) During the bone resorption phase, mature osteoclasts create resorption pits, known as Howship’s lacunae, on the bone surface. Osteoclasts release matrix metalloproteinases (MMPs), along with other enzymes, to degrade both the organic and inorganic components of the bone matrix. This process is essential for removing old or damaged bone tissue, thereby preparing the site for subsequent bone regeneration. By clearing the bone surface, osteoclasts create favorable conditions for osteoblasts to deposit new bone matrix, ensuring the continuous renewal and maintenance of healthy bone tissue over time (Manolagas, 2010). 3) In the reversal phase, osteoblasts begin to gradually form bone structures. During this phase, there is an increase in apoptosis of osteoclasts, and paracrine signaling molecules, such as transforming growth factor-β (TGF-β), promote the aggregation of osteoblasts to the site of bone defects, thereby promoting bone formation. 4) During the osteogenesis phase, osteoblasts synthesize and secrete osteoid, a bone matrix that gradually replaces damaged or lost bone tissue at the defect site. This osteoid undergoes mineralization to restore the structural integrity and strength of the bone. Key regulators, including Wnt signaling, sclerostin, and parathyroid hormone (PTH), play critical roles in this process. These factors promote osteoblast differentiation and enhance their activity, thereby stimulating and supporting the bone formation process to ensure effective repair and regeneration. 5) During the termination phase, the organic matrix undergoes mineralization, ultimately forming new bone tissue. Concurrently, a subset of osteoblasts differentiates into bone lining cells, which play a role in bone surface maintenance, while others undergo apoptosis. The newly formed bone tissue then transitions into a phase of continuous equilibrium remodeling, adapting gradually to mechanical loads. This process is sustained by the dynamic balance between bone resorption by osteoclasts and bone formation by osteoblasts, ensuring the structural strength and integrity of bone over the long term (Pignolo et al., 2021). Osteoporosis develops when this normal bone remodeling process is disrupted, often due to factors such as aging, estrogen deficiency, or lack of physical activity, leading to an imbalance between bone resorption and formation. This results in decreased bone density and increased fragility, making bones more susceptible to fractures (Figure 1).

### 2.2 Reactive oxygen species

Reactive oxygen species (ROS) is a term encompassing highly oxidizing molecules such as superoxide anion (O₂⁻), hydrogen peroxide (H₂O₂), and hydroxyl radicals (·OH^−^), which are by-products of mitochondrial electron leakage during the electron transport chain in aerobic respiration. While ROS play essential roles in cellular signaling and homeostasis, their high oxidative activity can cause significant cellular damage if not effectively neutralized by the body’s antioxidant defense systems. This imbalance, known as oxidative stress, contributes to various pathological conditions. Reactive oxygen species are involved in cell proliferation, survival, metabolism, apoptosis, and differentiation, and play an important role in bone regeneration. ROS are both important signaling molecules and potential damage factors. At lower concentrations, ROS act as signaling molecules that regulate key cellular processes such as proliferation, differentiation, and migration. In bone tissue, ROS influences bone remodeling and resorption by modulating the activity of osteoblasts and osteoclasts through various transcription factors and signaling pathways, including NF-κB, HIF-1α, and redox-sensitive mechanisms. Research has indicated that an appropriate level of ROS can support the proliferation and differentiation of osteoblasts, thereby accelerating bone tissue regeneration. They promote bone matrix production through activation of the Wnt/β-catenin pathway. Therefore, regulation of ROS levels is essential for promoting bone regeneration and preventing oxidative stress.

**FIGURE 2:**
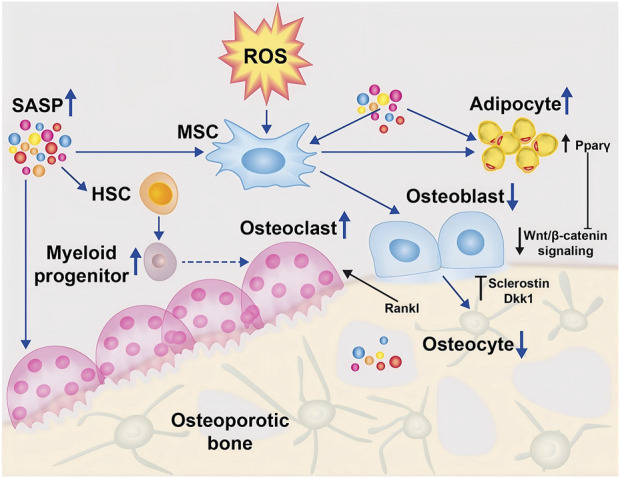
ROS regulates osteoblasts (Riegger et al., 2023). In the osteoporotic bone microenvironment, senescence-associated secretory phenotype (SASP) is increased and induces differentiation of mesenchymal stem cells (MSCs) into adipocytes through activation of the peroxisome proliferator-activated receptor gamma (Pparγ) pathway, leading to accumulation of adipocytes in the bone marrow. Indirectly, the differentiation of osteoblasts was reduced, further leading to deficient osteogenesis. In addition, SASP promotes the development of myeloid progenitor cells in the hematopoietic stem cell (HSC) lineage, which further promotes the increase of monocytes, thereby transiently increasing osteoclast formation. Furthermore, due to the balancing act between osteogenesis and osteoblastogenesis, osteoclast apoptosis induces the production of osteocalcin and Dickkopf-related protein (Dkk)1, which further inhibits osteogenesis by suppressing Wnt/β-catenin. (Open Access Copyright ^©^ 2024 BioMed Central Ltd unless otherwise stated. Part of Springer Nature).

ROS can also be produced by enzymes such as nicotinamide adenine dinucleotide phosphate oxidase (Nox1), cyclooxygenase, and lipoxygenase in response to external cellular stimuli, including growth factors. These enzyme-driven pathways contribute to ROS generation beyond mitochondrial activity, playing roles in cellular signaling and pathological processes (Janssen-Heininger et al., 2008). First, excess ROS upregulate multiple inflammation-related signaling pathways. For example, activation of the nuclear factor kappa-light-chain-enhancer of activated B cells (NF-κB) signaling pathway promotes the translocation of NF-κB dimers to the nucleus. This process occurs through the phosphorylation and ubiquitination of the inhibitor of NF-κB (IκBα), leading to its degradation. Once in the nucleus, NF-κB initiates the transcription of various genes, resulting in the production of pro-inflammatory cytokines (Zhang et al., 2016), chemokine, MMPs and other inflammatory mediator expression (Yu et al., 2022). At the same time, the body has a defense system against ROS, and a dynamic balance is maintained between the two. This system includes enzyme- and transcription factor-dependent antioxidant enzymes and cellular autophagy, the former being the expression of superoxide dismutase, catalase (CAT), glutathione peroxidase, and antioxidant genes induced by the transcription of forkhead transcription factor O isoforms (FoxO), etc. ROS activates the c-Jun amino-terminal kinase (JNK) pathway, phosphorylating FoxO transcription factors and releasing them from the cytoplasm into the nucleus (van der Horst and Burgering, 2007). ROS can activate the c-Jun N-terminal kinase (JNK) pathway, leading to the phosphorylation of the FoxO transcription factor and its translocation from the cytoplasm to the nucleus. After entering the nucleus FoxO was able to activate antioxidant enzymes such as manganese superoxide dismutase (MnSOD) and catalase (CAT). This process helps prolong the cell’s resting phase, protecting quiescent cells from oxidative damage and maintaining cellular homeostasis (van der Horst and Burgering, 2007). However, excessive ROS activation of FoxO transcription impairs limited β-catenin transcription to T cell transforming factor/lymphoid enhancer factor (TCF/LEF) and reduces osteoclastogenesis. Therefore, under normal conditions, the former alone is not sufficient to counteract intracellular damage due to ROS, and the cell’s own autophagy repairs or removes dysfunctional organelles and proteins due to excessive intracellular ROS through the ubiquitin-proteasome or lysosomal pathways to ensure further defense of cellular health (Manolagas, 2010). Oxidative stress occurs when the balance between ROS production and ROS removal is disrupted by increased mitochondrial damage and weakened defense mechanisms due to aging and disease, etc. ROS modulates multiple signaling pathways through activation or inhibition of various cytokines and enzymes, and upregulation or downregulation of receptor and ligand expression, which ultimately affects the expression of genes in the nucleus and promotes apoptosis of Bone mesenchymal stem cells (BMSCs), osteoclasts and osteoblasts and proliferation and differentiation of osteoblasts, resulting in a delay in the rate of bone formation relative to the rate of bone resorption, leading to a disruption of bone reconstruction. apoptosis of BMSCs, osteoblasts, osteoclasts, and osteoclast proliferation and differentiation, resulting in a lag in the rate of bone formation relative to the rate of bone resorption, leading to a disruption of the bone remodeling homeostasis (Camici et al., 2007). Under physiological conditions, ROS production is typically regulated by antioxidant defense systems, which maintain cellular redox balance. Key components of these antioxidant defense systems include vitamins E and C, glutathione peroxidase, reduced glutathione, superoxide dismutase (SOD), and catalase. These molecules work together to neutralize excessive ROS, thereby preventing oxidative stress and safeguarding cellular structures from damage (Parascandolo and Laukkanen, 2019). When intracellular ROS levels are carefully regulated, they function as second messengers, influencing and activating various signal transduction pathways. These pathways play vital roles in biological processes such as apoptosis, cell survival, differentiation, proliferation, and inflammation. Proper control of ROS signaling is critical for preserving cellular homeostasis and ensuring coordinated physiological responses (Ray et al., 2012). The rapid generation of ROS in response to RANKL stimulation plays a crucial role in the induction of osteoclast precursors, indicating that ROS functions as intracellular mediators in the differentiation of osteoclasts. This balance can be disrupted by oxidative stress, which is caused by certain oxidative pathways that lead to excessive ROS and make it difficult for the antioxidant system to maintain balance. This further leads to a loss of bone mass, which can lead to osteoporosis. Excessive ROS production significantly increases osteoclast formation while reducing the production and activity of osteoblasts. This imbalance contributes to alterations in bone structure and bone loss, hallmark features of osteoporosis (Kimball et al., 2021) (Figure 2).

### 2.3 Macrophage

Chronic inflammation plays a critical role in the pathogenesis of osteoporosis. Macrophage colony-stimulating factor (M-CSF) and granulocyte macrophage colony-stimulating factor (GM-CSF) play pivotal roles in directing the differentiation of monocytes into distinct macrophage subsets. Specifically, M-CSF promotes the differentiation of monocytes into the anti-inflammatory and immunosuppressive macrophage phenotype (M2), while GM-CSF drives the formation of the pro-inflammatory macrophage phenotype (M1) (Lukic et al., 2017). Macrophage polarization is the process by which the cellular surroundings, cytokines, and molecular signals differentially affect macrophages to differentiate into different cellular phenotypes. For example, classically activated M1 proinflammatory macrophages or alternatively activated M2 tissue wound-healing macrophages (Martinez and Gordon, 2014). In M1 macrophages, arginine metabolism is shifted to nitric oxide (NO) and citrulline; in M2 macrophages, arginine metabolism is shifted to ornithine and polyamines. It has been shown that M1-produced NO is a major effector molecule with the ability to inhibit cell proliferation and microbicidal activity, whereas M2-produced ornithine promotes cell proliferation and repair through polyamines and collagen synthesis, fibrosis and other tissue remodeling functions (Pesce et al., 2009).

Pro-inflammatory cytokines secreted by M1 macrophages stimulate osteoclast activity and enhance subsequent bone resorption. Macrophages are induced to polarize to M1 type by interferon-γ (IFN-γ), lipopolysaccharide (LPS), and tumor necrosis factor-α (TNF-α) (Savitri et al., 2020), and secrete TNF-α, interleukin-6 (IL-6), and interleukin-1 (IL-1), ROS and other cytokines (Bai et al., 2020), which have antibacterial and antitumor functions and promote reactive oxygen species-induced tissue damage. During bone metabolism, TNF-α upregulates target genes such as RANK through the NF-κB pathway and promotes osteoclastogenesis, and TNF-α inhibits osteogenic factors such as Runt-related transcription factor 2 (RUNX2) and osteoblast differentiation. In addition, M1 plays an important role in matrix destruction and tissue reorganization of damaged tissues by producing a variety of enzymes, such as MMP, collagenases, elastases, and hyaluronidases. This allows M1 to rapidly move through damaged tissue to remove pathogens and debris (Kou and Babensee, 2011). M1 macrophages are the main producers of TNF-α, and the long-term high level of TNF-α in chronic inflammation leads to the development of osteoporosis (Fernández et al., 2017).

**FIGURE 3:**
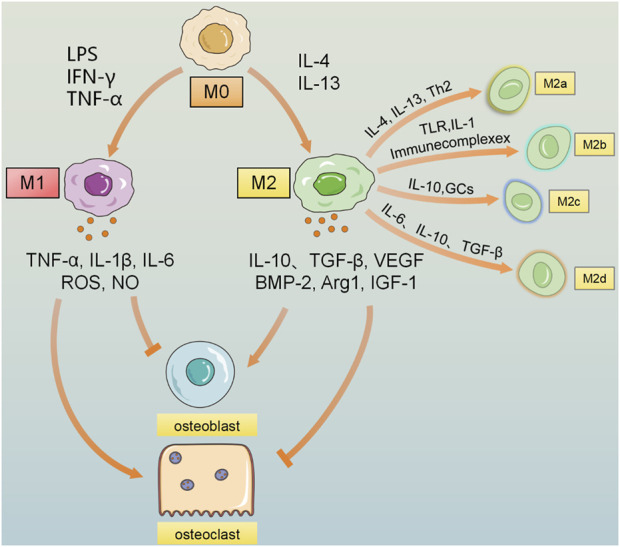
Macrophages are polarized to the M0 state, and under the induction of stimuli such as LPS, IFN-γ, and TNF-α, they are polarized to the pro-inflammatory M1 macrophages, secreting inflammatory factors such as IL-1β, IL-6, IL-12, TNF-α, NO, and ROS. M0 is polarized to the anti-inflammatory M2 macrophages under the stimulation of factors such as IL-4 and IL-13, etc., polarizes into the anti-inflammatory M2 macrophages. Under the stimulation of substances such as Th2 cells and immune complexes, it releases a large amount of anti-inflammatory IL-10, TGF-β, vascular endothelial growth factor (VEGF), etc. M2 can be further divided into 4 subtypes under the stimulation of different factors: M2a activated by IL-4 and IL-13, M2b induced by immune complexes, TLR, and IL-1, M2c activated by IL-10 and glucocorticoids, and M2d cell subtype polarized by factors such as IL-6, IL-10, and TGF-β. Due to the effects of different cytokines, the influence of M1 and M2 macrophages on bone remodeling is that M1 macrophages increase the formation of osteoclasts and bone resorption, leading to bone loss; on the contrary, M2 macrophages promote the proliferation and differentiation of osteoblasts, thereby contributing to bone repair and reconstruction and promoting the recovery of bone density.

M2 macrophages are capable of releasing various cytokines with anti-inflammatory effects such as interleukin 4 (IL-4), interleukin-10 (IL-10), interleukin-13 (IL-13), etc. In addition, M2 secretes a number of factors associated with the promotion of tissue repair and antioxidant activity, which contribute to the further polarization of the M2 cell phenotype. This positive feedback loop helps maintain an anti-inflammatory environment, supporting tissue repair and regeneration while counteracting inflammatory processes (Murray, 2017). Recent studies have shown that M2-polarized macrophages play a pivotal role in bone regeneration by inducing the differentiation of BMSCs into mature osteoblasts (Zhang et al., 2017). In addition to their anti-inflammatory effects, M2 macrophages further promote bone mineralization and stimulate the production of factors such as bone morphogenetic protein 2 (BMP-2), matrix metalloproteinase-9 (MMP-9), transforming growth factor β (TGF-β) and insulin-like growth factor 1 (IGF-1). These molecules are essential for promoting osteoblast differentiation and supporting bone formation. The mechanism is to upregulate osteogenic factors including RUNX2, alkaline phosphatase (ALP), and type I collagen (COL1) to promote osteogenesis. M2 can be further divided into M2a, M2b, and M2c. The M2a phenotype is associated with a T helper 2 cell (Th2) response and is produced in response to IL-4 and IL-13. The M2b phenotype is induced by immune complexes and Toll-like receptor (TLR) or IL-1 receptor agonists and secretes high levels of IL-10 but reduced IL-12. IL-10 or glucocorticoids induce the M2c phenotype, produces high levels of IL-10 and TGF-β, and is associated with immunosuppression and remodeling (Kou and Babensee, 2011; Garg et al., 2013). Notably, M2 macrophages produce significantly higher levels of BMP-2 compared to M0 or M1 macrophages. BMP-2 significantly promotes the differentiation of BMSCs to osteoblasts, a process that dramatically enhances the osteogenic potential of bone marrow mesenchymal stem cells, making BMP-2 a key factor in bone regeneration and repair (Gong et al., 2016) (Figure 3).

### 2.4 Osteoclast

Osteoclasts, the resident macrophages of bone tissue, are situated on the bone surface, where they play a central role in bone resorption. In osteoporosis, their activity is heightened, leading to excessive bone degradation and contributing to localized inflammation, which exacerbates the condition and disrupts normal bone remodeling processes (Yang and Yang, 2019). During osteoclast differentiation, RANKL first activates bone marrow-derived macrophages (BMM), which further initiates the NF-κB and MAPK signaling pathways. This activation drives osteoclastogenesis by upregulating the expression of key osteoclast lineage-associated genes, including c-Fos, Mmp9, cathepsin K, and nuclear factor of activated T cells cytoplasmic 1 (NFATc1). These genes are critical for the maturation, function, and resorptive activity of osteoclasts (Teitelbaum and Ross, 2003). Pro-inflammatory cytokines can activate osteoclast activity and promote bone resorption both directly and indirectly (Souza and Lerner, 2013). For example, IL-1 indirectly promotes bone resorption by increasing the production of RANKL in osteoblasts. RANKL subsequently binds to RANK receptors on osteoclast precursors, driving their differentiation and maturation into functional osteoclasts (Park et al., 2017). M2-polarizing cytokines, such as IL-4 and IL-13, have been shown to decrease bone resorption by suppressing osteoclast precursor differentiation and inhibiting the activity of mature osteoclasts. While much of the research on macrophages, inflammation, and bone loss has focused on reducing osteoclast activity to counteract inflammation and bone degradation, chronic inflammation driven by M1 macrophages remains a significant contributor to osteoporosis. Anti-inflammatory cytokines can mitigate this by inhibiting osteoclast function and bone resorption, presenting a promising therapeutic strategy. Thus, promoting M2 macrophage polarization through specific cytokines may offer an effective approach to improving bone health in osteoporosis patients.

### 2.5 Treatment and prevention of osteoporosis

#### 2.5.1 Medication

There are currently three main classes of drugs used to treat osteoporosis: antiresorptive drugs, anabolic drugs and selective estrogen receptor modulators (SERMs). Anti-metabolic or anti-resorptive drugs inhibit or attenuate osteoclast activity and promote osteogenesis to maintain bone homeostasis, thereby preventing bone loss and increasing bone strength. However, long-term use of antiresorptive drugs disrupts the natural repair of bone tissue and affects the balance between the osteogenic and osteoblastic systems. Over the past 2 decades, substantial advancements have been achieved in this therapeutic field, leading to the development of numerous chemical and biological agents that effectively reduce the risk of vertebral fractures and, in some instances, non-vertebral fractures, including hip fractures. While treatments are now available for glucocorticoid-induced osteoporosis and male osteoporosis, there is still ongoing research to further improve outcomes for these conditions (Kanis et al., 2013), the most significant progress in osteoporosis treatment has been seen in postmenopausal women, where targeted therapies have substantially improved management. These advancements have focused on reducing fracture risk and improving bone density, helping to address the hormonal imbalances that contribute to bone loss in this population. Although anti-osteoporotic medications come with potential side effects, they offer additional health benefits, such as reducing the incidence of certain cancers and lowering overall mortality rates, alongside improving bone quality (Diez-Perez et al., 2012).

Osteoporosis therapeutic agents (e.g., bisphosphonates), although highly soluble, have low absorption, resulting in very limited oral gastrointestinal absorption and utilization. Moreover, in order to achieve therapeutic efficacy when used locally, it is often necessary to maintain a high dose to maintain the local drug concentration, which is more likely to cause serious local side effects (Chiu et al., 2020). To overcome these limitations, scaffold materials designed for localized drug delivery have emerged as a promising approach. Multifunctional hydrogel-based biomaterials, loaded with therapeutic agents, are increasingly utilized in osteoporosis treatment, enabling precise drug delivery to the affected area while minimizing adverse effects (Zheng et al., 2021). 3D printing technology enables the efficient creation of surface structures for drug delivery, offering enhanced control over the drug-carrying and release processes. This technology allows for more precise regulation of local drug concentrations, ensuring controlled and sustained release at the target site, which improves the overall effectiveness and minimizes side effects of treatment.

#### 2.5.2 Surgical treatment

Osteoporosis has a major impact on fracture healing. Effective fracture healing depends on the stability and osteogenic capacity of the fracture site. In the case of osteoporotic fractures, the reduction in bone mass and destruction of bone microstructure reduces the mechanical strength of the bone and increases the likelihood of fracture, especially in fractures with severe comminution of the trabecular region of the bone, where destruction of the trabecular structure not only dramatically increases the difficulty of surgical reset, but also has a significant impact on later healing. Biologically, BMSCs in osteoporotic bone exhibit a diminished capacity for osteogenic differentiation, likely due to reduced expression of BMP-2. Furthermore, osteoporotic fractures are often associated with impaired angiogenic potential, further limiting effective bone repair and regeneration. These combined factors underscore the complexity of managing fractures in osteoporotic patients. (Kwong and Harris, 2008).

The fracture fixation approach for osteoporotic bone is influenced by its unique mechanical properties. It is crucial to consider that even during routine activities, bones and joints experience significant and dynamic loads, with the magnitude and direction of these forces constantly changing. In order to address the stress mismatch between the contact surfaces of the bone and the implant that leads to surgical failure, internal fixation materials with lower rigidity and higher modulus of elasticity, such as intramedullary nails and tension bands, are often used. The lower stiffness gives these materials better flexibility to cope with the mechanical strength of bone tissue in osteoporotic conditions (Beltran et al., 2016). Severe comminution complicates fracture repositioning, and large bone defects do not allow for regeneration of bone tissue and reconstruction of bone structure between normal bone tissues without intervention, which usually requires surgical grafting of human bone tissue or biomaterials to fill the bone defects. To achieve optimal stability and facilitate efficient fracture healing, a combined strategy involving the use of implants and biomaterials is frequently adopted. This strategy not only enhances mechanical stability but also supports biological processes crucial for bone repair and regeneration.

The spine, hip, and distal radius are the most common sites of fractures associated with osteoporosis. Surgical techniques for managing these fractures have advanced considerably in recent years. Distal radius fractures, often termed “sentinel fractures,” are frequently the first clinical indication of underlying osteoporosis. Current evidence suggests that surgical interventions, particularly using palmar or dorsal plating approaches, provide superior outcomes compared to conservative management. In cases involving severe comminution and collapse of bone fragments, biomaterials are commonly employed to fill bone defects, stabilize the fracture, and enhance healing, leading to improved patient outcomes.

## 3 3D printed biomaterials

Large bone defects caused by bone trauma, infection, osteoporosis, arthritis, obesity, diabetes and cancer remain a major problem in orthopaedics and can cause damage to bone tissue leading to non-healing, bone tissue defects, severe pain and deformity (Agarwal and García, 2015). Bone tissue is highly capable of self-healing within a certain range of defects, but if the defect reaches a certain level (usually >2 cm), the bone cannot heal completely without external intervention (Mouriño and Boccaccini, 2010). Autologous bone grafting has been regarded as the gold standard for treating bone defects (Hassani et al., 2005). However, the limited availability of autologous bone poses a challenge and can result in additional complications, such as morbidity associated with donor-site healing. Biocompatible and bioactive bone substitute implant materials are expected to address the bottleneck in the clinical application of bone substitutes compared to autologous bone (Mueller et al., 2011).

Biomaterials have achieved a wide range of clinical applications, non-biological materials, such as metals, ceramics, and polymers, are mainly used in bone tissue engineering as scaffolds, fillers, and fixation devices to support bone repair and regeneration (Qin et al., 2024). Titanium and titanium alloys have been widely used in bone repair scaffolds, joint implants and bone fixation devices. Their mechanical properties are stable and their greater inertness makes them very difficult to be affected by the external environment. Calcium phosphates such as hydroxyapatite used as bone filler materials have similar inorganic composition to bone, are highly biocompatible and have been widely used in dental and orthopedic clinics (Zhang et al., 2024). The main problems with non-biological materials are difficulty in matching the degradation rate to the bone regeneration process, potential foreign body reactions after implantation, and the need for more long-term clinical efficacy and safety data. Biomaterials include natural biomaterials and bioactive factors, which are commonly used to promote bone cell proliferation, differentiation and angiogenesis. Collagen, as a bone tissue scaffold material, has been widely used for bone defect repair, especially in dentistry and craniofacial surgery. Gelatin can be used as a cellular scaffold material, which can effectively promote osteoblast adhesion and differentiation. Bone morphogenetic proteins (BMPs) have been approved by the FDA for bone defect repair.BMP-2 and BMP-7 are the most common in the clinic (Dubey et al., 2021). However, although biomaterials have better bioactivity, there are some existing problems such as the possible release of cytotoxic products during degradation, and challenges in mass production and consistency control of biomaterials (Yuan et al., 2024) (Table 1).

**TABLE 1:** Clinical applications of non-biomaterial and biomaterial systems.

Material type	Representative material	Applications and features	Limitation	References
Non-biological material	Metal	Stable mechanical properties as bone repair scaffolds, joint implants, internal fixation devices	May trigger an inflammatory response and prolonged implantation may result in the release of metal ions	Fan et al. (2024), Yang et al. (2017)
	Ceramic	High bioactivity, promotes osseointegration, as bone filler material, bone graft substitute	Higher brittleness, difficult to withstand large mechanical loads	Fernandes et al. (2017)
	Polymer	Degradation rate can be controlled, easy to process, and can be used as biodegradable scaffolds and bone fillers by compositing multiple materials	Insufficient mechanical properties, may produce acidic degradation products	Bharadwaz and Jayasuriya (2020)
Biomaterials	Natural biomaterial	Biocompatible, promotes cell adhesion and proliferation as a scaffold for cartilage and bone repair	Degradation rate is difficult to control and may trigger an immune response	Sorushanova et al. (2019), Guimarães et al. (2022)
	Bioactive factor	Promote osteoblast differentiation and new bone formation, with bone defect repair, bone fusion effects	High cost of use, possible release of cytotoxic products during degradation, some materials are difficult to mass produce	Dubey et al. (2021), Shen et al. (2022)

The early use of autologous bone grafts and allogeneic bone grafts suffered from greater limitations due to factors such as donor and surgical trauma, so bone substitutes with biocompatibility and bioactivity have a wider range of application prospects. Bone implant materials are critical for cell proliferation and differentiation into their surface. Early techniques used for scaffold fabrication include solvent casting, gas foaming, phase separation, emulsion freeze drying, solution casting, and freeze drying (Wüst et al., 2011). These traditional methods have many limitations because they often cannot create precise pore size, pore geometry, high levels of interconnectivity, and high mechanical strength (Do et al., 2015). 3D printing has been developed as an advanced technology to overcome the limitations of these methods and produce scaffold materials that can more effectively promote bone tissue regeneration.

3D-printed complex bone biomaterials exhibit a high degree of similarity to human bone tissue in both macro and microstructures. This makes them particularly well-suited for combining with active substances such as cells and growth factors, enhancing their potential for effective bone tissue reconstruction. Such advancements are especially valuable for the personalized treatment of bone defects. Meanwhile, the integration of 3D printing technology with multidisciplinary fields such as tissue engineering, digital medicine, and materials science has led to the development of 3D printed products with excellent biocompatibility, strong osteogenic induction capabilities, and stable mechanical properties. In clinical applications for bone defect repair, a variety of biomaterials and 3D printing techniques are utilized to fabricate patient-specific bioactive scaffolds. The microstructure of these scaffolds is carefully engineered to meet the complex demands of bone defect repair, enabling personalized treatment approaches tailored to individual patient needs. As an ideal bone replacement material, key factors such as high porosity and excellent cytocompatibility are critical for success. Unlike traditional machining methods, additive manufacturing technology builds structures by adding material layer by layer, allowing for greater design flexibility and precision (Derby, 2012).

### 3.1 Tissue engineering with 3D printed biomaterials

Tissue Engineering is an interdisciplinary field that combines biology, materials science and engineering, aiming at constructing, repairing or replacing damaged or dysfunctional tissues and organs (Sun et al., 2022). Tissue engineering has undergone decades of development and a considerable number of materials related to tissue engineering have been developed, some of which have been used in clinical applications, such as a variety of metallic materials for repairing and filling bone defects and polymer skin dressings for promoting skin healing. These materials are applied explicitly to various tissues, such as bone tissue engineering, cartilage tissue engineering, vascular tissue engineering and neural tissue engineering (Zhu et al., 2016). Recent advances in materials research have greatly supported tissue engineering, while 3D printing technology offers precise and personalized manufacturing, allowing bioscaffolds to replicate the structure and function of natural tissues more effectively. The core of tissue engineering is cells, biomaterials and bioactive factors, which complement each other to promote tissue regeneration. 3D printing materials offer a level of precision unmatched by conventional materials, enabling the accurate delivery of drugs or bioactive factors. By controlling the shape, size, and porosity of tissue engineering scaffolds, they allow precise cell implantation and regulate interactions between cells and materials, promoting the synergy of multiple factors to achieve tissue repair and regeneration (Kelly et al., 2018). Factors synergize to achieve tissue repair and regeneration. The development of 3D printing materials and the full integration of multiple fields have enabled existing materials to exhibit excellent biocompatibility, osteoinductivity, and mechanical properties (Arif et al., 2022).

The main challenges in today’s tissue engineering include matching the degradation rate of materials with the efficiency of tissue regeneration, the ability to promote vascularization well, and immune rejection. Degradability is a key feature of 3D-printed biomaterials, helping to minimize the long-term impact of foreign bodies. Some materials release substances during degradation that promote tissue regeneration. However, if the degradation is too fast or too slow, it can disrupt the tissue repair process. The angiogenic ability of biomaterials directly affects the local circulation and metabolism of tissues, and good angiogenic ability is a key factor for biomaterials to promote tissue repair at the macro level (Wei et al., 2024). Future advancements in 3D-printed biomaterials focus on developing smart materials that respond to external stimuli, combining multiple materials for diverse functionalities, and integrating stem cell technology to enable multidirectional differentiation and construct complex local structures (Alonso-Fernández et al., 2023).

### 3.2 3D printing technology

Bio-3D printing is an important form of technology that assembles biomaterials by layer-by-layer deposition with computer-assisted deposition, and also allows for the precise colonisation of cells on bioscaffolds by adjusting the shape, size and porosity of the tissue engineering scaffolds, which can be used to regulate the interaction between the cells and the material (Kim et al., 2017). 3D printing technology can mimic the microenvironment and biological components of the organism using suitable biomaterials and cell types to create functional 3D structures. 3D printing technology makes it easy to prepare scaffolds with specially designed dimensions, porosity and interconnecting channels that favour cell growth (Duan et al., 2010). The main 3D printing technologies commonly used in orthopaedics are cured stereolithography (Zhou et al., 2023; Zhang et al., 2019a; Kafle et al., 2021), selective laser sintering (Koons et al., 2020/08; Sears et al., 2016), fused deposition modelling (Abdullah et al., 2018; Ligon et al., 2017), inkjet printing (Huang et al., 2021), etc. Three-dimensional printing can be categorized into low-resolution printing, where the accuracy is typically <100 μm, and high-resolution printing, where some extrusion methods fall into this category. The latter precision can be maintained stably below 100 μm, including some of the higher precision requirements of the technology such as stereolithography, selective laser sintering and inkjet printing. Each approach provides unique benefits in terms of resolution, material compatibility, and printing speed, enabling customized solutions tailored to the specific needs of various applications. The materials include metal materials, ceramic materials, polymer materials and composite materials. 3D printing also increases the fit of the material to the bone tissue, increasing the contact area and providing good conditions for bone tissue regeneration (Table 2).

**TABLE 2:** Different types of 3D printing techniques.

3D printing techniques	Advantages	Disadvantages	References
Extrusion-based 3D Printing	1. More compatible materials 2. Adjustable pore size 3. Convenience and low cost	1. Low resolution 2. Increased material shear due to the extrusion process	Kafle et al. (2021), Jiang et al. (2020)
Selective laser sintering	1. Powdered materials 2. High resolution 3. High material utilization	1. High temperatures affect the biological activity of materials	Kamboj et al. (2020), Brunello et al. (2016)
Inkjet printing	1. High resolution 2. Multi-material simultaneous printing 3. Low cost	1. Higher material viscosity required 2. Lower stability	Huang et al. (2021), Tharakan et al. (2021)
Low-Temperature 3D Printing	1. Maintaining biological activity 2. Precise control of the porous structure	1. Lower mechanical strength 2. Acidic degradation products	Wubneh et al. (2018), Dadhich et al. (2021)

#### 3.2.1 Extrusion 3D printing technology

3D printing via extrusion is currently the most widely used and earliest technology for biomaterials, with high adaptability and low work requirements, capable of processing most materials, including thermoplastics, thermosets, hydrogels, ceramics, and more. These materials have non-Newtonian shear-thinning behavior, appropriate viscosity, high mechanical strength and thermoplasticity, and controlled structural strength based on printability. These characteristics ensure that the materials can flow smoothly during printing while maintaining structural integrity and functionality after the printing process (Jiang et al., 2020). The material is extruded as a continuous filament through a nozzle driven pneumatically or mechanically. The extruded material is deposited onto a platform to create a two-dimensional structure. By moving the nozzle or platform along the *z*-axis, a layer-by-layer deposition process is used to build the desired 3D structure. This method allows for the creation of scaffolds with layered architectures, varying pore sizes, and high cell densities, making extrusion-based 3D printing an effective technique for producing complex and functional biomaterials (Wang J. et al., 2021). While extrusion-based 3D printing offers relatively low resolution compared to other techniques, it supports a wide range of printable materials and enables the fabrication of tissue structures with enhanced mechanical strength. Its ability to accommodate customizable pore sizes, achieve high cell densities, and maintain a relatively low cost makes it the preferred method for producing biomaterial scaffolds for osteoporosis treatment. This method allows for precise control over scaffold structure and properties, making it an ideal choice for creating functional scaffolds that promote bone regeneration and repair in osteoporotic patients (Su et al., 2018). In this way, numerous composite scaffolds for tissue engineering have been developed, such as polyurethane/poly (lactic acid)/graphene oxide (TPU/PLA/Go) (Chen et al., 2017), polycaprolactone/hydroxyapatite (PCL/HA) (Shor et al., 2007), and hydroxyapatite/poly (ester-urethane) (HA/PEU) (Yu J. et al., 2017).

#### 3.2.2 Selective laser sintering

Selective laser sintering (SLS) uses materials in the form of fine powders (10–100 μm) such as metal, ceramic or polymer powders (Kamboj et al., 2020). The materials are bonded to each other as they reach their melting point in the presence of a laser. SLS requires particles to exhibit good flow dynamics within the system, which is typically achieved through surface functionalization to minimize electrostatic forces. This process ensures that the particles flow smoothly during sintering, allowing for the precise formation of the desired structure and enhancing the overall quality of the final product (Guvendiren et al., 2016). Selective laser sintering (SLS) is capable of handling more complex material shapes and structures, and the complexity of bone tissue defects makes SLS a good candidate for application. However, since plasticized materials almost always rely on their thermosetting or thermoplasticity to be printed, the high temperatures during the printing process can negatively affect the bioactivity and effectiveness of biological factors, drugs, etc., added or modified in the material, which can have an uncontrollable effect on the outcome of the final printed material. This challenge requires careful consideration of material selection and printing parameters to ensure that the therapeutic properties of the scaffolds are preserved.

#### 3.2.3 Inkjet printing

Inkjet printing uses electricity or heat to power a nozzle that ejects bio-ink (e.g., hydrogel) in tiny droplets that are deposited layer by layer to form a three-dimensional structure. This technique allows for high precision in placing bio-inks, making it suitable for creating intricate scaffolds with controlled cell distribution and layer formation (Huang et al., 2021). Inkjet printing allows very small amounts (1–100 pL) of individual droplets to be ejected from the nozzle onto the print surface (Derby, 2010), where the structure is formed after curing. Inkjet printers are divided into two categories: continuous inkjet (CIJ) printing and on-demand inkjet (DOD) printing. Depending on the droplet generation mechanism, in CIJ printing, a continuous stream of droplets (approximately 100 µm in diameter) is generated. In DOD inkjet printing, individual droplets (in the range of 25–50 µm in diameter) are generated when required (Derby, 2010).

The resolution of inkjet printing is between 25 and 50 μm (Derby, 2010). Inkjet printing can mimic the porous structure and mechanical properties of bone trabeculae at higher resolutions (Vanderburgh et al., 2017). In addition to its high resolution, inkjet printing requires materials with specific viscosity characteristics, typically below 10 cP. While low viscosity allows for precise droplet formation, it can also lead to challenges such as poor material stability and increased degradation. These issues may affect the structural integrity and long-term functionality of the printed scaffolds, making material selection and optimization critical for successful applications in tissue engineering and bone regeneration (Guvendiren et al., 2016). Additionally, the mechanical or thermal stress exerted on cells during the inkjet printing process can result in cell damage, which poses a limitation to the broader application of this technology (Tharakan et al., 2021). Barak and Black (2018) replicated bone trabeculae of the same structure using 3D printing and developed a standardised model of the trabecular structure (using different thicknesses of trabeculae according to different segmentation thresholds) for the treatment of postmenopausal osteoporosis.

**FIGURE 4:**
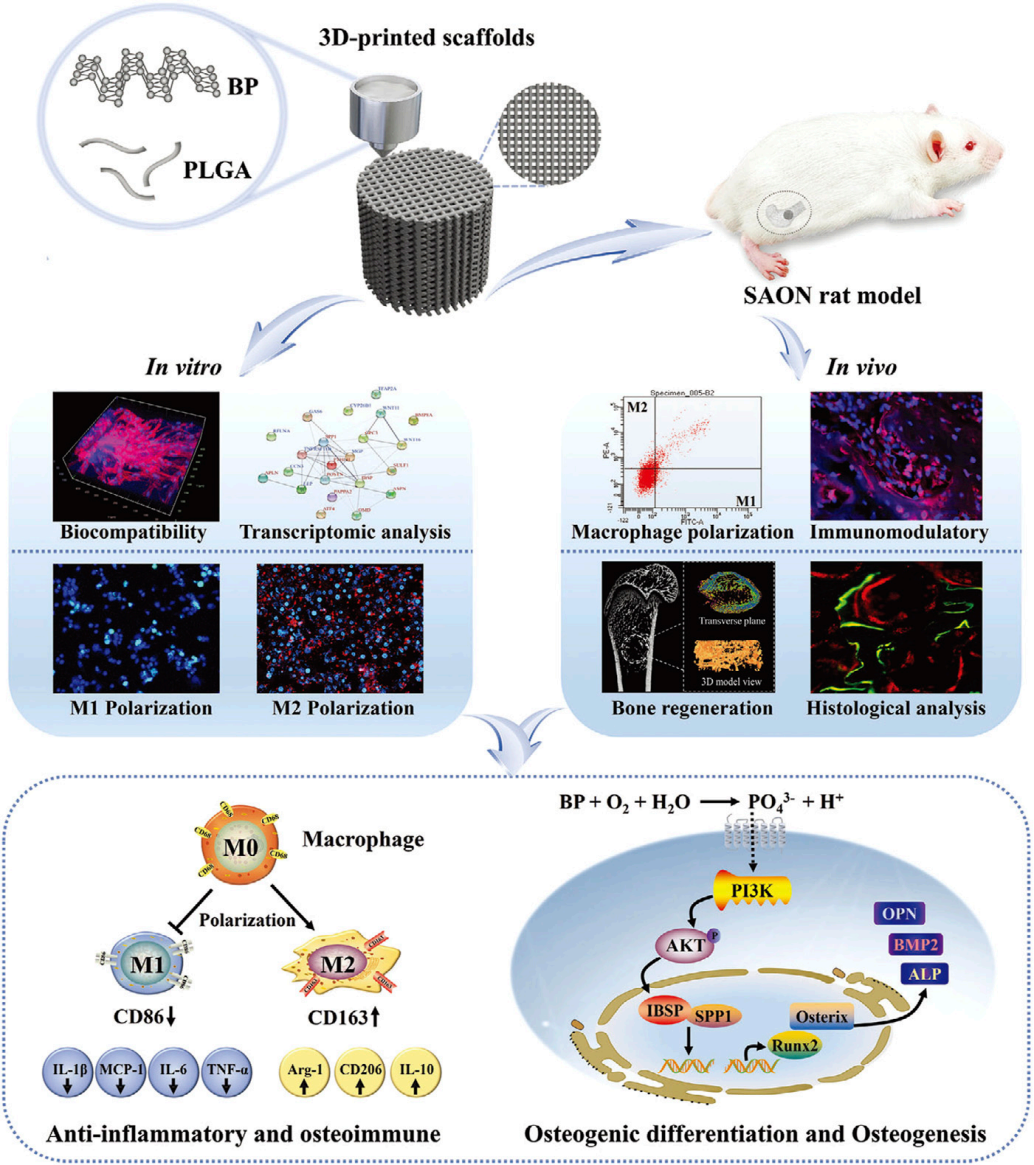
The PLGA/BP scaffold manufactured by 3D printing is associated with the mechanism of BP degradation inducing an osteoimmune environment to accelerate bone regeneration. It was found that the PLGA/BP scaffold can recruit and stimulate macrophage M2 polarization, inhibit inflammatory responses, and promote the proliferation and differentiation of BMSCs in *in vitro* and *in vivo* experiments, thereby promoting bone regeneration in the femoral distal defect area of a steroid-associated osteonecrosis (SAON) rat model. In addition, PLGA/BP scaffolds were screened and shown to promote osteogenic differentiation through transcriptome analysis, and it was verified that PLGA/BP scaffolds can promote osteogenic differentiation of human BMSCs by activating the PI3K-AKT signaling pathway (Long et al., 2023). (Reproduced with copyright ^©^ 2023 Long J, Yao Z, Zhang W, et al. Advanced Science published by Wiley‐VCH GmbH).

Serious complications, including displacement between implant and bone, contact surface loosening, and peri-implant fracture, are one of the greatest challenges associated with the use of bone implants for osteoporotic diseases (Liu et al., 2016; Quan et al., 2018). Metallic materials such as titanium alloys have high mechanical strength and corrosion resistance and are currently the most widely used materials for orthopaedic implants (Wang B. et al., 2021), but most of them are too hard and may lead to stress shielding-induced osteolysis (Li J. et al., 2019). 3D printed porous titanium (pTi) scaffolds can be significantly reinforced and printed to meet the desired shapes and surface areas (Nune et al., 2016).3D printing can also help personalise osteoporosis treatment and predict fracture risk. In addition, it can help treat osteoporosis by creating bone trabeculae with incredible structural properties (Figure 4).

#### 3.2.4 Low-temperature 3D printing

Low-Temperature 3D Printing is a technology designed for cellular and tissue materials, allowing printing at reduced temperatures. This preserves the activity and structural integrity of biomaterials while avoiding the negative impact of high temperatures on bioactivity seen in conventional printing methods (Geven and Grijpma, 2019/06). Low-temperature conditions can lead to more stable material properties, and computer modeling can be used to more finely construct the porous structure of the material as well as control the porosity (Liu et al., 2017). In this method, the required material is typically in liquid form. Techniques like inkjet printing, stereolithography, and extrusion molding operate at room temperature, where the material is rapidly cooled through the nozzle or mold to solidify under low-temperature conditions. Subsequently, lyophilization is used to remove the solvent, separating it from the material and creating a porous structure as a result. In addition, the avoidance of high temperatures allows for the maintenance of the biological activity of some drugs, hydrogels, chitosan and other materials that are not resistant to high temperatures (Qin et al., 2021).

### 3.3 3D printing biomaterials classification

#### 3.3.1 Polymers

Polymeric materials can be categorized into natural polymers, such as gelatin, chitosan, and hyaluronic acid; and synthetic polymers, such as polycaprolactone (PCL), polylactic acid (PLA), polyglycolic acid (PGA), and poly (lactic-co-glycolic acid) (PLGA). Natural polymers offer excellent biocompatibility, while synthetic polymers provide good degradability. Some synthetic polymers also feature controlled degradation rates and mechanical strength, making them suitable for the structural needs of bone scaffolds (Koh et al., 2019; Verné et al., 2015).

PLA is a synthetic linear aliphatic polyester with good mechanical properties (Backes et al., 2021), biocompatibility and degradability, with high modulus of elasticity and structural strength, enabling it to have strong mechanical properties as a bone implant material (Akmal et al., 2018). But PLA is inert, has a low rate of degradation, and degradation can produce acidic products, which can lead to localized inflammatory reactions. PCL is also a synthetic biodegradable aliphatic polymer. It has excellent properties such as good biocompatibility, modulus of elasticity and biodegradability, and is widely used in clinical applications (Backes et al., 2022). However, PCL is relatively stable in living organisms and does not show any significant degradation within 6 months, affecting local bone regeneration. Alam F et al. Doping of tricalcium phosphate (TCP) and hydroxyapatite (HAp) into PCL-based scaffolds improves bone regeneration and osteoinductive properties (Alam et al., 2020/01). PLGA is a copolymerization of PLA and polyethylene glycol (PEG), a hydrophilic polymer, and PLA, a hydrophobic polymer, and the hydrophilicity of PLGA can be adjusted by adjusting the ratio of the two materials. Degradable polymers have the ability to be metabolized and absorbed by the body (Schliecker et al., 2003).

#### 3.3.2 Metals

Metals have been widely used in bone tissue engineering due to their excellent mechanical properties, easy processing, and structural stability. Currently, common metal scaffold materials include cobalt-nickel alloys, stainless steel, and titanium alloys. However, higher hardness often means lower elasticity modulus. In osteoporotic conditions, bone tissue has reduced structural strength compared to normal, and conventional metallic materials may cause stress shielding due to the large difference in strength between the material and bone tissue, which can significantly hinder bone tissue regeneration (Liverani et al., 2021). The results showed that 3D printed Ti scaffolds facilitated the adhesion, proliferation, mineralization and differentiation of preosteoblasts (Nune et al., 2015). Zhong W et al. prepared a Ti scaffold using 3D printing and surface modified it with polydopamine to obtain a good hydrophilic surface and increase the surface binding ability of the implant to the bone (Zhong et al., 2020). Tantalum and niobium metal surfaces are characterized by high friction properties, high porosity, and moderate mechanical properties, but they are expensive and difficult to process, and the application of 3D-printed tantalum and niobium scaffolds is limited. Therefore, tantalum and niobium metals can be used as coatings for surface modification of traditional metal materials to improve the surface properties of traditional metal materials while saving costs. Recently, the excellent degradability of magnesium metal has received much attention. However, magnesium has insufficient corrosion resistance in the internal environment of human body fluids and usually loses its proper mechanical strength prematurely.

#### 3.3.3 Inorganics

In the field of bone regeneration, inorganic materials are of interest due to their similarity to natural bone tissue, excellent mechanical properties and bioactivity. 3D printing technology allows these materials to be precisely constructed into scaffolds with complex geometries and porosities, thus promoting bone tissue regeneration and repair more effectively. Common inorganic materials include Bio-ceramics, Tricalcium Phosphate (TCP) and Bioactive Glass (BG). Bio-ceramics include Hydroxyapatite (HA) (Feng et al., 2017/11), Biphasic Calcium Phosphate (BCP), Magnesium Phosphate (MCP) etc. (Saffarzade et al., 2020/04), which have excellent biocompatibility and biodegradability. HA is commonly used for bone defect repair, and 3D printing technology allows for complex processing of Hydroxyapatite. Fitzpatrick V et al. utilized 3D printing to produce silk Hydroxyapatite scaffolds with complex structures. The scaffold material has suitable mechanical properties, good cytocompatibility, and osteoinductive properties. It can maintain bone cell morphology and cytokine activity, indicating that hydroxyapatite has potential to promote bone regeneration (Fitzpatrick et al., 2021). β-Tricalcium phosphate (β-TCP) is a CaP-based ceramic that is osteoconductive and biodegradable, resembling the mineral phase of natural bone tissue, and is used to build porous structures by 3D printing technology to provide space for cell adhesion and angiogenesis. However, it lacks optimal mechanical stability to withstand large loads. BG is able to bind to bone tissue without rejection. When BG is used on bone tissue, it forms carbonated hydroxyapatite on its surface, which promotes the regeneration of bone tissue (Jones, 2013). And the components of BG promote the differentiation and maturation of BMSC (Hench and Jones, 2015). 3D printing technology can also create BGs with hollow or mesoporous structures, allowing the incorporation of drugs or metal ions. This synergistically enhances their ability to promote bone regeneration (Wu et al., 2013; Quinlan et al., 2015).

#### 3.3.4 Bioactive agent

Protein-activated factors and peptides are important bioactive agents in osteoporosis treatment, which regulate bone metabolism through a variety of biological mechanisms, thereby promoting bone regeneration and reducing bone resorption. Protein-activated factors (e.g., BMPs and VEGF) enhance the production of bone matrix by stimulating the proliferation and differentiation of osteoblasts (Dubey et al., 2021), and promote vascularization to provide nutritional support for bone repair. Peptides (e.g., the RANKL inhibitor deslizumab and the parathyroid hormone analog teriparatide) (Ouyang et al., 2021), on the other hand, reduce bone resorption or promote new bone formation by inhibiting osteoclast activity or intermittently activating osteoblasts. The former is better suited for repairing large bone defects, while the latter has proven highly effective in treating systemic osteoporosis. Together, they have the potential for synergistic effects in clinical applications (Zhang et al., 2021).

### 3.4 Characterization and properties of 3D printed biomaterials

Usually, biomaterials used as bone implants have to fulfill several basic conditions, such as biocompatibility, adequate mechanical properties, suitable porosity and their surface structure (Codrea et al., 2021). Biocompatibility ensures that the metamaterial itself does not trigger a severe inflammatory response, while adequate mechanical properties are able to withstand the loads of normal movement and can be vascularized to obtain a normal bone structure (Pereira et al., 2020). Porosity and the surface properties of biomaterials are crucial factors in promoting bone regeneration. Pores within the material facilitate the entry of cells, creating a large internal surface area that supports cell attachment and provides diffusion sites (Amini et al., 2012), this structural feature is essential for enhancing cell infiltration, nutrient exchange, and overall tissue integration, which are key to successful bone healing and regeneration. The porosity of the material promotes cell infiltration and vascularization, thus providing a means of nourishment to the nascent tissue. All of these parameters affect the transport of nutrients and oxygen throughout the scaffold. They also influence cell-material interactions, which play a critical role in regulating bone regeneration. Properly designed scaffolds with optimized porosity and surface features ensure effective nutrient and oxygen supply to the cells, enhancing their function and promoting successful bone tissue formation. A variety of materials have been 3D printed to promote bone regeneration, and combinations of natural and synthetic materials, such as calcium phosphate (CaP) and PCL, have been shown to improve cell adhesion and aid bone growth (Serra et al., 2013). PCL is widely used in 3D printing because it can be processed by melting and does not require the use of solvents.

#### 3.4.1 Surface factors of 3D-printed biomaterials

Surface topography of biomaterials has a significant effect on cell behavior such as cell adhesion and cell migration (Bacáková et al., 2001; Dave and Gomes, 2019). Surface roughness increases the anchoring force of the implant on the tissue and does not limit its attachment and spreading (Vagaská et al., 2010). In the field of bone regeneration biomaterials, the significance of surface roughness in bone repair and regeneration has been extensively studied, particularly on metallic surfaces such as titanium alloys. The roughness of the metal surface can be easily changed by mechanical means (such as surface sandblasting) or chemical treatment (such as acid etching and anodizing) (Kim et al., 2008). Bone implant materials with certain surface roughness have been shown to have a superior ability to promote osseointegration compared to titanium implants with smooth surfaces (Buser et al., 2004). Cell culture well plate surface roughness also affects the behavior of osteoblasts and promotes the differentiation of BMSCs towards the osteogenic spectrum (Kunzler et al., 2007). Since the natural extracellular matrix (ECM) in the biological microenvironment is organized in a specific conformation, surface roughness plays a crucial role in cellular colonization. The main biomechanical effects of surface roughness on cells include changes in cell shape and modifications in integrin signaling. These changes impact osteogenic differentiation by modulating the interaction between cells and the scaffold surface, potentially enhancing or impeding bone regeneration depending on the material’s roughness and structural design (Olivares-Navarrete et al., 2015). D-printed biomaterials can be engineered with precise microstructures, including grooved, columnar, porous, or more intricate geometries, to optimize cell behavior and improve bone regeneration outcomes (Unadkat et al., 2011).

Artificially mineralizing a material can improve surface roughness. For example, when calcium phosphate biomineralizes a scaffold, the arrangement of calcium phosphate crystals increases the hydrophilicity and surface roughness of the scaffold, which significantly improves its biocompatibility and cellular interactions for bone regeneration. This modification improves the scaffold’s ability to promote cell attachment, infiltration, and osteogenic differentiation, contributing to better bone regeneration outcomes (Fiedler et al., 2013). The biomineralized portion of the scaffold mimics a bionic matrix that provides contact guidance for cells, influencing their behavior. This structure not only promotes the osteogenic differentiation of UCMSCs but also enhances alkaline phosphatase activity, a key marker of osteoblast function. By facilitating both cellular orientation and differentiation, biomineralization contributes significantly to improved bone regeneration outcomes (Li W. et al., 2018). Bruyas A et al. conducted a study in which PCL and β-tricalcium phosphate (TCP) scaffolds were 3D printed to develop bionic implants, offering a potential solution for disc replacement. The results showed that a higher proportion of β-TCP led to increased surface roughness, which in turn enhanced cellular activity. This increased roughness facilitated better cell attachment and proliferation, promoting the potential for improved tissue integration and healing in the context of disc replacement (Bruyas et al., 2018).

Surfactants have been reported to significantly affect integrin binding specificity and regulate cell differentiation (Keselowsky et al., 2005). Surface silylation (Cicuéndez et al., 2014; Collart-Dutilleul et al., 2014) and alkanethiol modification (Chieh et al., 2013; Bai et al., 2013) approaches have been introduced to surface functional groups for cellular studies, and functionalization with amines, hydroxyls, and carboxyls on surfaces significantly upregulates osteogenic differentiation. Plasma vapor deposition has gained attention as an alternative approach for surface modification of bone tissue scaffolds, offering precise control over surface properties to enhance their biological performance (Vasudev et al., 2013). It does not require a solvent process and does not use any liquid chemicals, nor does it require special surface chemistry to form the coating. Research by Yu, W et al. has shown that plasma polymerization can generate coatings rich in amine and carboxyl functional groups, thereby improving osteogenesis on 3D scaffolds (Yu et al., 2020; Griffin et al., 2016).

#### 3.4.2 Mechanical properties of 3D printed biomaterials

As a bone implant biomaterial, elasticity and stiffness also affect the growth of regulatory cells. Studies have shown that the elasticity of a material is closely linked to its stiffness, which can influence the differentiation of BMSCs. For example, the modulus of elasticity of some metallic materials is much greater than that of the existing bone tissue, so the load on the bone tissue around the metallic implant is reduced, resulting in a stress-shielding effect (Huiskes et al., 1992). This results in prolonged low stress levels in the surrounding bone tissue, depriving it of adequate mechanical stimulation, which can worsen conditions like osteoporosis and even lead to implant detachment. Therefore, selecting a material with an appropriate modulus of elasticity is crucial for effective bone regeneration and maintaining implant stability. Polyacrylamide gels were used and crosslinked with different concentrations of a bis-crosslinking agent to simulate elasticity. The study showed that acrylamide helps to change elasticity. Collagen-coated polyacrylamide provides adhesion. Studies on materials with adjustable stiffness have demonstrated that increased stiffness enhances the differentiation of naïve BMSCs into osteoblasts (Chaudhuri et al., 2020). Further studies investigated the impact of viscoelasticity by transplanting cells into hydrogels with identical initial moduli of elasticity but varying stress relaxation rates. Hydrogels with faster stress relaxation rates were found to enhance bone regeneration more effectively, highlighting the importance of viscoelastic properties in guiding cellular behavior and improving tissue repair outcomes (Darnell et al., 2017).

The elastic modulus of a material plays a crucial role in influencing osteoblast adhesion, proliferation, and differentiation. A lower elastic modulus typically supports osteoblast adhesion and spreading, while a higher elastic modulus tends to promote osteoblast differentiation, directing cells toward bone regeneration. This highlights the importance of tailoring the elastic modulus of biomaterials to optimize different stages of bone tissue formation (Turnbull et al., 2018). 3D printing technology can be used to design 3D printed bone regeneration-promoting biomaterials with different elastic modulus by using a variety of materials or gradient structures to mimic the heterogeneous structure of natural bone better. The hardness and flexibility of the materials can be optimized at different sites to meet the mechanical needs of different regions of the bone tissue.

The Rheology and Injectability of the material determines the processability during printing, printing accuracy, and functionality of the final scaffold or tissue (Ustunel et al., 2024). Rheology refers to the fluidity and deformation behavior of the material under external force, and commonly used parameters include viscosity, elasticity, etc. In the unformed stage, the material prepared by heating or using liquid solvents should have low viscosity to allow smooth extrusion or plasticization. It should also quickly maintain its shape during the molding and curing stages to achieve the desired mechanical properties (Tien and Dance, 2021). Injectability, on the other hand, refers to the performance of being able to smoothly extrude through a tiny nozzle and maintain continuity without clogging under the action of external forces (Monia et al., 2022). Extrusion-based 3D printing remains the most commonly used method. Injectability directly affects the feasibility of material printing, and the extrusion process does not damage the material at the composition or structural level. Common injectable materials include gelatin and alginate. In practice, the rheology of the material often determines its injectability, requiring a balance between high fluidity for smooth extrusion and shape stability after printing (Hua et al., 2021).

#### 3.4.3 Pore size and porosity of the material

The porous structure of biomaterials plays a vital role in facilitating cell attachment, growth, proliferation, migration, and differentiation. For bone implants, critical parameters such as porosity, pore connectivity, and pore size must be carefully considered to ensure optimal cellular interactions and support for bone regeneration. Pore connectivity is particularly important for allowing nutrient flow, cell growth, and gas transport within the scaffold. Porosity and pore size in the macrostructure of a material can significantly affect the aggregation and activity of cells, and can determine whether the cells are able to access the nutrients normally required by the material (Mandal and Kundu, 2009). If the pore size in the scaffold is too small, it can hinder cell migration and lead to tissue cell necrosis due to inadequate nutrient and oxygen supply. On the other hand, if the pores are too large, the overall surface area of the material is reduced, which limits cell adhesion and proliferation (Murphy et al., 2013). Pore size is essential for ensuring the efficient delivery of nutrients and the removal of cellular waste. Consequently, an ideal 3D-printed biological scaffold should exhibit high porosity to optimize nutrient exchange, waste elimination, and cell infiltration, thereby supporting effective tissue regeneration.

Different cells and tissues have their own appropriate porosity for optimal growth environments. For example, fibroblasts have the highest proliferative activity in pore sizes between 186–200 μm, and osteoblasts and chondrocytes have very strong proliferative and differentiation properties in larger pore sizes of 380–405 μm. Additionally, scaffolds with pore sizes between 290 and 310 μm demonstrate a faster rate of new bone formation compared to scaffolds with pore sizes outside this optimal range, highlighting the importance of tailoring pore size for specific regenerative applications (Oh et al., 2007). Scaffolds with 325 μm pores have been shown to support a greater number of osteoblasts and enable faster cell migration. Pore sizes of 100 μm are relatively more suitable for cell adhesion and proliferation, while pore sizes larger than 325 μm allow for a high degree of cell mobility and a significant increase in cell migration. Consequently, the optimal pore size must be carefully tailored to meet the specific requirements of the tissue being regenerated, balancing cell attachment and migration to achieve effective tissue growth and regeneration (Murphy et al., 2010).

For bone tissue engineering, Using a freeze-drying method based on (3,4-ethylenedioxythiophene) and poly (styrene sulfonate), Guex AG et al. prepared a highly porous poly-scaffold, most notably characterized by pore connectivity, with an average pore size of 53.6 ± 5.9 μm.This scaffold was effective in facilitating the infiltration of MC3T3-E1, with significant calcium deposition and extracellular matrix formation at 28 days (Guex et al., 2017). In a three-dimensional scaffold of silk fibroin based on a cryogenic process of freeze-drying of a hydrogel, the porosity and pore size can be further controlled and adjusted by changing the freezing temperature and freezing rate. The maximum pore size is 200–250 μm at −20°C, and the porosity is 86% for the 3D scaffold with a pore size of 80–100 μm and a porosity of 96% at −196°C. Cell proliferation experiments showed that even in the small pore scaffold at −196°C, cell proliferation was significantly promoted, indicating that the porosity of the material ultimately determines the proliferation capacity of cells (Mandal and Kundu, 2009).

#### 3.4.4 Fidelity

Fidelity refers to the degree to which the structure of the printed material resembles the designed model in terms of morphology, size, and functionality. High fidelity means that the printed biological scaffold or tissue can accurately reproduce the complex structure of the digital model and fulfill the intended biological function. Fidelity can be categorized into structural fidelity, morphological fidelity, cellular fidelity, and functional fidelity (Nelson et al., 2021). Factors that can affect fidelity include printing resolution, material curing rate, material biocompatibility, material degradation rate, etc. Several methods can improve material fidelity, such as increasing the viscosity to reduce collapsing during printing, using a small-diameter nozzle to enhance resolution while minimizing cellular damage, employing rapid cross-linking techniques like light curing, and using double cross-linking, where physical curing is followed by chemical curing (Landau et al., 2025). The fidelity of 3D-printed biomaterials is a crucial factor influencing their biological performance. It can be greatly improved by optimizing material properties, adjusting printing parameters, and enhancing the cross-linking process.

#### 3.4.5 Degradability

**FIGURE 5:**
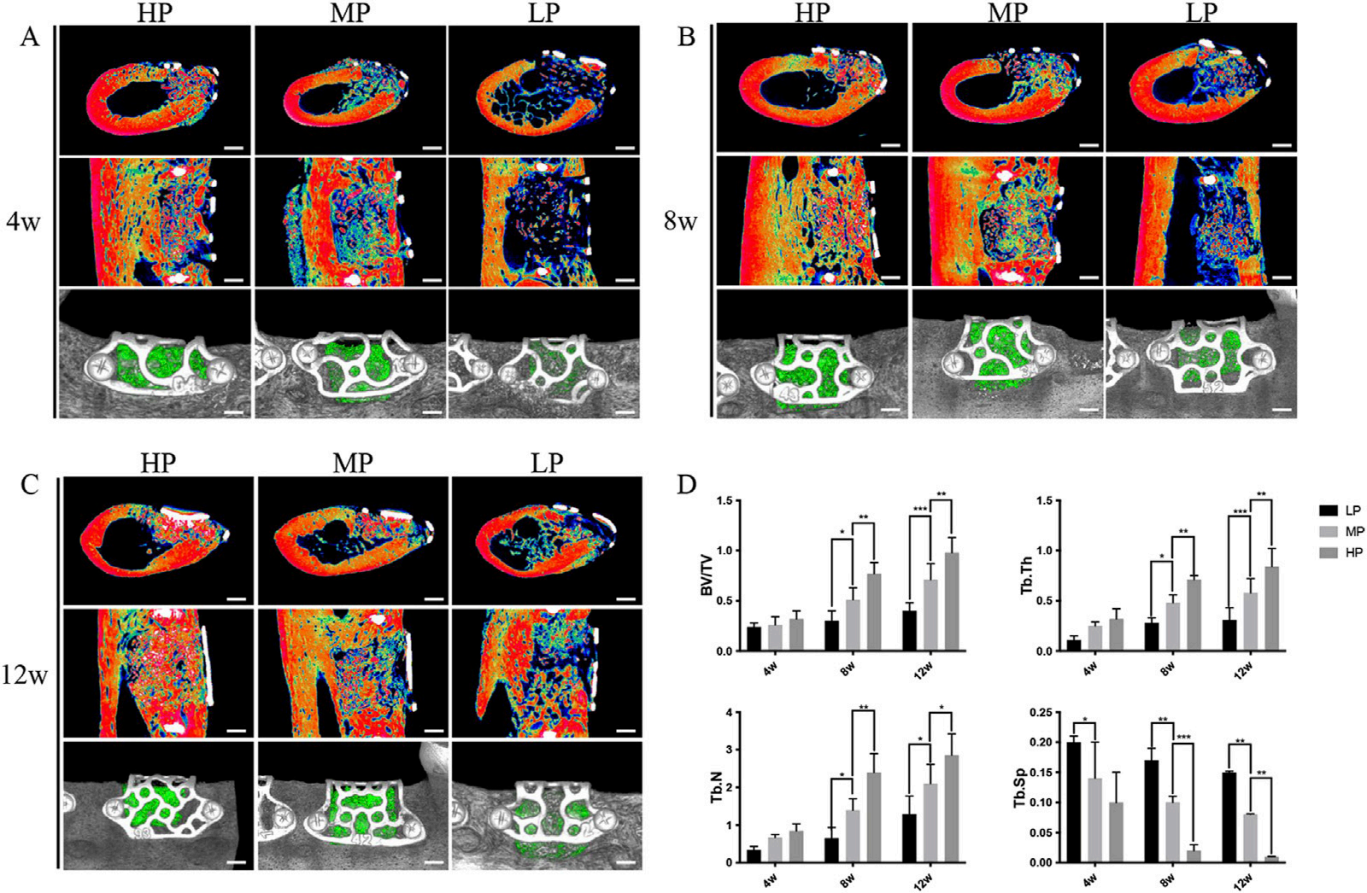
Micro-CT imaging results demonstrating the effect of titanium mesh with varying porosities on bone defect repair. Titanium meshes with low porosity (LP) 55%, medium porosity (MP) 62%, and high porosity (HP) 68% were prepared using laser sintering technology, followed by surface sandblasting. **(A)** At 4 weeks post-implantation, significant new bone formation was observed in the HP, MP, and LP groups, with the HP group showing the most notable effect. **(B)** By 8 weeks post-implantation, all groups exhibited further bone defect repair compared to 4 weeks, with density and volume changes in new bone consistent with earlier observations. **(C)** After 12 weeks of implantation, the HP group demonstrated the most substantial bone repair effect compared to the other groups. **(D)** Parametric analysis revealed that trabecular thickness (Tb. Th), trabecular number (Tb. N), and bone volume fraction (BV/TV) increased as healing time progressed, while trabecular separation (Tb. SP) decreased. The density of new bone continued to show an upward trend throughout the healing period. These findings indicate that higher porosity titanium scaffolds promote more effective bone regeneration over time. (Ma et al., 2023). [Reproduced with Open Access from Ma, R., Liu, Q., Zhou, L. et al. High porosity 3D printed titanium mesh allows better bone regeneration. BMC Oral Health 23, 6 (2023)].

Some 3D printed biomaterials are able to gradually break down into small molecules or harmless products in the *in vivo* or *in vitro* environment and are absorbed or excreted by tissues and cells. The degradation process must align with the rate of tissue repair and regeneration to ensure new tissue fills the defect area as the material degrades. If the degradation is too fast, the biomaterial may not provide enough support if the bone defect has not gained sufficient strength. Conversely, if the degradation is too slow, the biomaterial may remain in the defect area for too long, hindering the proliferation of new bone tissue (Nelson et al., 2021). Degradation can be classified into several types: hydrolytic degradation, which is influenced by the material’s chemical structure; enzymatic degradation, regulated by enzymes; biodegradation, where the material degrades through biochemical reactions and the products are safely metabolized by the organism; and solvation degradation, where the material degrades in solvents, primarily depending on the solvent’s pH value. In order to regulate the degradability of a material, the rate can be adjusted by combining materials with different degradation rates; adjusting the crosslinking density of a material can also increase or decrease the degradation process of the material (Choe et al., 2022/07). In addition, in recent years, there are also some responsive materials, which are capable of degrading in specific environments (e.g., pH, temperature), and regulating degradation processes more precisely. PLGA/β-TCP composite scaffolds can gradually degrade at the site of bone defects while promoting new bone generation. Li C et al. doped osteoinductive Zn^2+^ into PLGA/β-TCP using cryomolding technology, which was able to release a safe dose of Zn^2+^ within 16 weeks, facilitating the growth-in and osteogenic differentiation of BMSCs (Li et al., 2023) (Figure 5).

A number of studies in recent years have shown that cells adhere, grow and proliferate more on a circular scaffold than on an orthogonal scaffold. For bone tissue engineering, scaffolds with non-orthogonal geometries are better suited to replicate the natural extracellular matrix structure. In a study conducted by Fonseca DR et al., 3D-printed PCL scaffolds with 56% porosity and a distinctive circular and sinusoidal design were used to seed human osteosarcoma cells (Saos2). This innovative design aimed to enhance the biomimetic properties of the scaffold, supporting improved cell interactions and tissue regeneration. The study assessed the viability and proliferation of the Saos2 cells cultured on these scaffolds. The results showed a significant difference between cells cultured on non-orthogonal scaffolds and those on orthogonal scaffolds after 21 days. The non-orthogonal geometry significantly enhanced Saos2 mineralization and promoted calcium deposition in osteogenesis experiments. Mechanical property testing revealed that scaffolds with sinusoidal geometry exhibited the highest elastic modulus in both dry and wet conditions. Additionally, non-orthogonal geometries outperformed orthogonal geometries in terms of elastic modulus, highlighting their superior mechanical performance for applications in bone tissue engineering (Fonseca et al., 2018). The incorporation of graphene oxide (GO) into 3D-printed scaffolds has been shown to enhance their porosity. This reduces the reliance on traditional techniques, such as solvent casting, that are typically used to increase scaffold porosity. Graphene oxide not only improves the porosity but also enhances the mechanical properties and bioactivity of the scaffolds, making them more suitable for applications in bone tissue engineering (Boga et al., 2018).

Scaffolds constructed with reduced graphene oxide (RGO) demonstrate high porosity, exceeding 90%, making them well-suited for bone tissue engineering. However, increasing the concentration of nanohydroxyapatite (nHA) within these scaffolds from 20% to 80% reduced the porosity to approximately 86% ± 1.5%. This finding highlights the substantial influence of nHA content on the porosity and overall structural development of porous scaffolds. BMSCs exhibited higher adhesion on RGO-prepared 20% nHA@RGO scaffolds, indicating that a moderate concentration of nHA in the scaffold enhances cellular attachment. However, increasing the nHA content in the scaffolds beyond this concentration can negatively impact porosity and alter cell-cell interactions. Higher nHA loading may lead to toxicity and growth inhibition in the cells, likely due to reduced porosity and compromised nutrient and oxygen transport (Nie et al., 2017/05). High porosity 3D printed scaffolds have a significant effect on promoting angiogenesis in bone tissue in addition to stimulating cell growth and migration (Mehdizadeh et al., 2013). The addition of HA in 3D printing materials affects porosity, pore connectivity, as well as bone mineralization and bone healing properties. Gleeson JP et al. added 200 weight percent (wt%) of HA to collagen-based scaffolds, which resulted in scaffolds with a porosity of 99%, and the use of MC3T3-E1 with scaffolds that showed mineralization on both the 14th and 21st day of *in vitro* incubation, along with their stiffness and pore connectivity by approximately tenfold (Gleeson et al., 2010).

In conclusion, effective treatment of osteoporosis requires materials with high porosity and large pore sizes to support cell growth, along with a low elastic modulus and high viscosity to match the mechanical properties of osteoporotic bone. Despite advancements in biomaterials research that have enhanced material properties, significant challenges remain. These include achieving an optimal balance between material porosity and mechanical strength and refining scaffold compositions and surface treatments to ensure sufficient porosity while enhancing bone regeneration capabilities (Table 3).

**TABLE 3:** Main parameters of 3D printed biomaterials for induced bone regeneration.

Characterization	Main parameters	Effects on bone regeneration	Typical material	References
Surface factor	Surface roughness, chemical functional groups, bioactive coatings	Improves cell adhesion, osteoblast differentiation, and promotes bone mineralization	Hydroxyapatite, bioglass, collagen coating	Yuan et al. (2024); Chen et al. (2024/12)
Mechanical property	Modulus of elasticity, compressive strength, tensile strength	Ensures mechanical stability, avoids scaffold collapse, and matches the mechanical properties of bone tissue	Polylactic acid (PLA), β-tricalcium phosphate (β-TCP)	Long et al. (2023), Serra et al. (2013), Chen et al. (2014)
Pore size and porosity	Pore size (100–500 μm), porosity (50%–90%)	Promotes cell migration, nutrient transport and angiogenesis	Polycaprolactone (PCL), gelatin-chitosan composites	Turnbull et al. (2018), Ma et al. (2023), Karageorgiou and Kaplan (2005)
Fidelity	The extent to which the printing process reproduces the details of the design model	Ensure accurate reproduction of the micro- and macro-structure of the scaffold to provide a stable biological microenvironment	Polylactic acid (PLA), gelatin-chitosan composites	Rajabi et al. (2021), Li et al. (2018b)
Degradability	Matching the rate of material degradation to the rate of bone regeneration	Degradation products are non-toxic and can be metabolized by the body, and the degradation rate is adapted to the bone tissue repair process	Polyglycolic acid (PGA), chitosan, calcium alginate	Agarwal et al. (2023), Beheshtizadeh et al. (2023)

### 3.5 Antioxidant 3D printed biomaterials

In ROS-mediated inflammatory diseases, the body’s physiological ROS scavenging systems often fail to counteract excessive ROS production effectively. Recently, biomaterials with specialized ROS scavenging properties have been developed, offering promising therapeutic potential for the treatment of inflammation-mediated conditions. ROS-scavenging biomaterials can be classified into three distinct categories based on their mechanisms of action: those that mimic or amplify enzymatic activity to degrade ROS via catalytic processes, those that interact directly with ROS to neutralize them, and those that inhibit ROS generation by targeting their source to suppress production.

#### 3.5.1 Neutralization of ROS

N-acetylcysteine (NAC) is a widely used antioxidant in clinical research and cell culture studies (Mu et al., 2021). It has been shown to mitigate bone mass loss, reduce osteoblast apoptosis (Shieh et al., 2019), alleviate oxidative stress, and suppress osteoclastogenesis following gonadectomy (Chen et al., 2019). Vitamin K, an anti-osteoporosis agent, plays a crucial role in enhancing bone strength and promoting osteoblast proliferation. Additionally, it reduces oxidative stress and the production of ROS. Studies have demonstrated that vitamin K protects cells from H₂O₂-induced alterations in protein expression and facilitates the formation, remodeling, and mineralization of bone tissue (Ambrożewicz et al., 2019). Moreover, metallic nanomaterials and nanoenzymes have been engineered to regulate the oxidative environment, offering a promising therapeutic approach for osteoarthritis management (Yin et al., 2015). Li J. et al. developed a 40 nm diameter gold nanoparticles conjugated with 2,2,6,6-tetramethylpiperidinyl-N-oxygen (TEMPO), which were effective in reducing the ROS level of BMSCs under H_2_O_2_ exposure conditions and could maintain the promotional effect on osteogenic differentiation of BMSCs (Li J. et al., 2017). However, their limited mechanical properties hinder their broader application in osteochondral regeneration (Zhong et al., 2019). Consequently, there is a pressing need to design antioxidant tissue-engineered scaffolds capable of simultaneously regulating both the biochemical and physical microenvironments in osteoporosis treatment.

#### 3.5.2 Eliminate ROS

Recent research has revealed that melanin possesses potent oxidative stress scavenging properties, offering protection to healthy tissues (Bao et al., 2018; Hong et al., 2020). Deng C et al. introduced a novel bioceramic scaffold fabricated using 3D-printed magnesium-aluminate-talcite (AKT), a distinctive bioceramic material with demonstrated osteogenic bioactivity. AKT has shown significant potential in enhancing bone growth and mineralization, positioning it as a promising candidate for bone tissue engineering applications (Deng et al., 2022; Li T. et al., 2019). The scaffold was further enhanced with hair-derived antioxidant nanoparticles (HNPs) or microparticles (HMPs) to create a composite material (HNP/HMP-AKT) designed to regulate the biochemical and physical microenvironment in osteoarthritis synergistically. This composite effectively scavenged ROS and promoted osteochondral regeneration. HNPs/HMPs activated the glucose transporter pathway (GLUT), while the scaffold significantly facilitated chondrocyte proliferation and maturation. Additionally, the AKT bioceramic scaffold markedly accelerated the osteogenic differentiation of BMSCs. This study highlights the potential of integrating antioxidant properties into biological scaffolds to elucidate their mechanisms in chondrocyte maturation, BMSC osteogenic differentiation, and *in vivo* bone cartilage regeneration. The findings suggest this approach as a promising strategy for bone-cartilage regeneration in the treatment of osteoporosis.

Bioceramic scaffolds have been widely investigated in bone tissue engineering, with numerous 3D-printed bioceramic scaffolds demonstrating favorable mechanical properties and bioactivity. Despite these advancements, their performance in osteoporotic environments characterized by elevated ROS levels is often neglected. Among these materials,β-TCP stands out as a commonly used bioceramic for bone grafting, owing to its superior osteoconductivity and biodegradability (Dorozhkin and Epple, 2002). The degradation products of β-TCP scaffolds, including calcium and phosphate ions, are critical in facilitating bone formation by enhancing osteoblast activity and promoting mineralization. These ions contribute to establishing a conducive microenvironment for bone healing and regeneration. Moreover, the incorporation of specific chemical modifiers into β-TCP scaffolds can further optimize their properties, enabling the development of tailored scaffolds for enhanced bone regeneration. Metal-organic frameworks (MOFs), a class of porous hybrid materials composed of metal ions or clusters coordinated with organic ligands, represent a promising avenue for functionalizing these scaffolds to improve their regenerative potential (Shyngys et al., 2021). By combining specific metal cations or clusters with organic ligands, MOFs can be designed to create catalytic active sites with broad-spectrum ROS scavenging capabilities. This property allows MOFs to effectively neutralize harmful ROS, which are often involved in various pathological conditions, including bone diseases like osteoporosis (Furukawa et al., 2013). And the degradation of MOF can release biologically active metal ions. A zinc-cobalt bimetallic Metal-Organic Framework (Zn/Co-MOF) has been found to exhibit catalytic activity that promotes the scavenging of ROS, functioning similarly to enzymes such as peroxidase (POD), catalase (CAT), and superoxide dismutase (SOD) (Bagheri et al., 2019). Zinc has been found to be the most effective enzyme for the development of ROS. Zinc is an essential trace element with notable antioxidant and anti-inflammatory properties, playing a critical role in regulating the formation of free radicals in the body (Chasapis et al., 2020). It has been shown to positively influence both angiogenesis and osteogenesis, making it a valuable component in promoting tissue regeneration and bone healing (Sun et al., 2021). Shu C et al. developed a β-TCP scaffold functionalized with a Zn/Co-MOF, which exhibited capabilities to scavenge ROS, regulate inflammation, and support osteochondral regeneration for repairing osteochondral defects. This modified scaffold effectively promoted osteogenic differentiation and chondrocyte maturation of BMSCs. Additionally, it offered protection against oxidative stress by neutralizing external ROS and establishing an anti-inflammatory microenvironment, making it a promising strategy for osteochondral defect repair (Shu et al., 2023). Manganese, present in bone at an average concentration of 1.7–3 ppm, is an essential trace element that plays a critical role in protein synthesis within bone tissue (Brodziak-Dopierała et al., 2013; Carluccio et al., 2020). Mn^2^⁺ ions enhance osteoblast adhesion, viability, and proliferation by activating integrins (Bracci et al., 2009). Materials containing Mn^2^⁺ ions have been shown to upregulate osteogenic gene expression and promote increased collagen deposition (Yu L. et al., 2017). Recent studies further demonstrate that manganese supplementation in ovariectomized rats increases bone density, stimulates bone regeneration, and prevents bone loss, indicating its potential as a therapeutic strategy for treating osteoporosis (Torres et al., 2014). Li J et al. introduced manganese into *β*-TCP to prepare Mn-TCP bioceramics, which possessed ROS scavenging capabilities. Mn^2+^ ions released from Mn-TCP bioceramics scavenged ROS by activating Nrf2 to inhibit osteoclast formation, promote osteoblast differentiation, and accelerate bone regeneration under osteoporotic conditions *in vivo* (Li J. et al., 2021). In addition, Magnesium and β-TCP-bound bioceramics have immunomodulatory immune microenvironment properties that favor osteogenesis (Chen et al., 2014). Thus, β-TCP scaffolds, which exhibit favorable mechanical properties and bioactivity, can serve as an ideal matrix for osteochondral scaffolds with antioxidant capabilities.

#### 3.5.3 Enzymatic reaction

**FIGURE 6:**
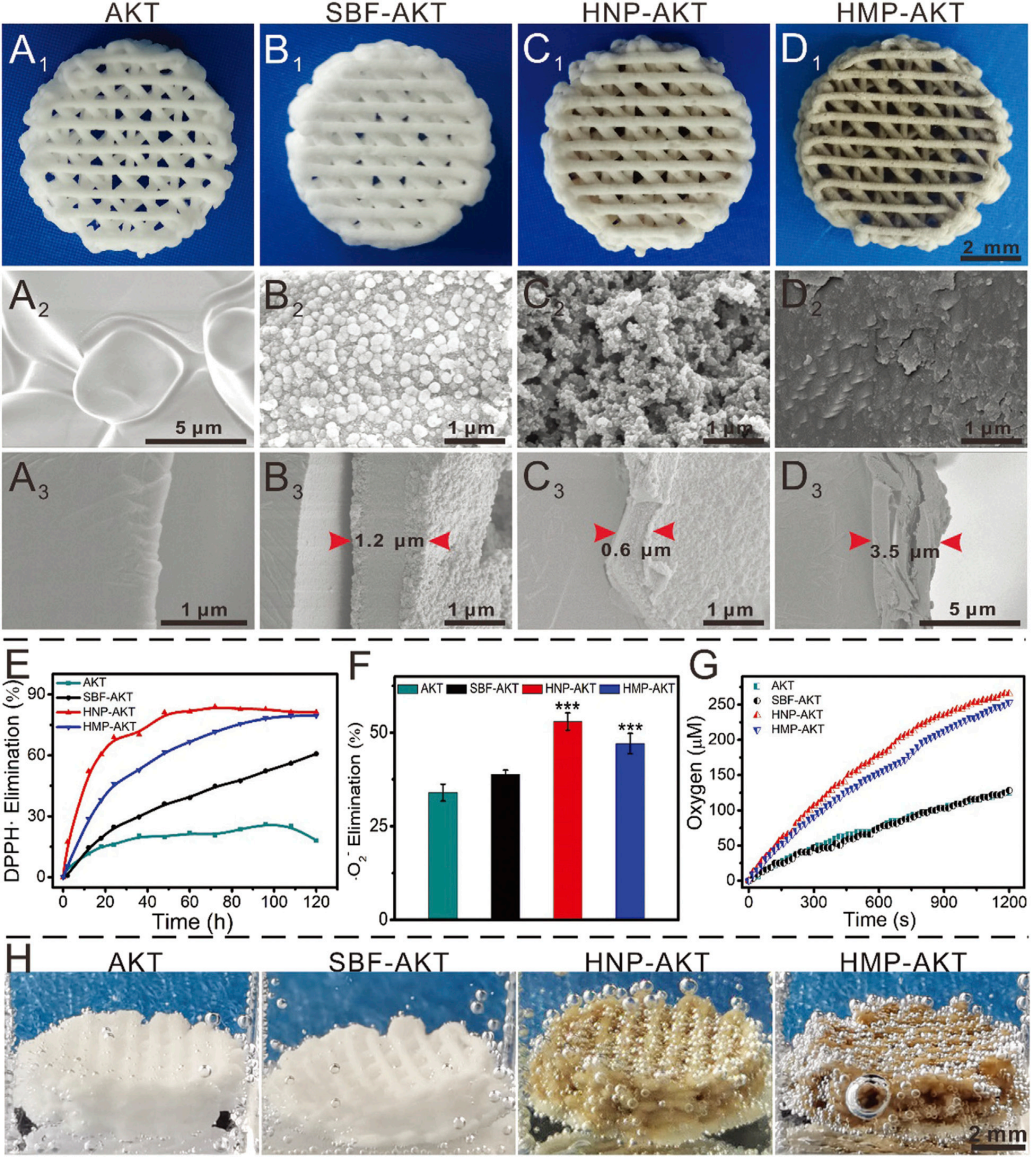
3D‐printed bioceramic scaffolds with integrated akermanite (AKT) and hair‐derived antioxidant nanoparticles (HNPs)/microparticles (HMPs) exhibit a multi‐enzymatic effect and can efficiently scavenge ROS (Deng et al., 2022). Photographs of **(A**_**1**_ **-D**_**1**_ **)** scaffolds demonstrate their physical appearance, while SEM images of **(A**_**2**_ **-D**_**2**_ **)** show detailed scaffold surface morphology, and SEM images of **(A**_**3**_ **-D**_**3**_ **)** reveal the cross-sectional structure. **(E)** Nitrogen free radical scavenging effect. **(F)** illustrates the superoxide radical scavenging effect, and **(G)** depicts oxygen production in the presence of 25 mM H₂O₂. Additionally, **(H)** captures images of oxygen vesicles formed by the material following H₂O₂ treatment. The HNP-AKT and HMP-AKT scaffolds exhibited remarkable scavenging activity against nitrogen radicals, superoxide anion radicals, and H₂O₂, demonstrating catalase-like (CAT) activity, which contributes to their potential for oxidative stress regulation and tissue regeneration. (Reproduced with Open Access from Deng C, Zhou Q, Zhang M, et al. Bioceramic Scaffolds with Antioxidative Functions for ROS Scavenging and Osteochondral Regeneration. *Adv Sci (Weinh)*. 2022; 9 (12):e2105727).

Introducing enzymes such as SOD and CAT into 3D printed scaffolds can reduce oxidative stress and promote cell proliferation and tissue repair. Bioactive glass (BG), a widely used ceramic biomaterial for bone repair, is known for its ability to form a layer of bone-like apatite on its surface *in vivo*. This process releases calcium and silica ions, which stimulate the formation of the bone matrix. However, BG’s clinical application is hindered by its high brittleness and insufficient modulus of elasticity. To address these limitations, incorporating inorganic nanoparticles into BG composites has emerged as an effective strategy to enhance its mechanical properties. Among these, cerium dioxide nanoparticles (CeO₂ NPs) stand out as potent antioxidants capable of scavenging ROS efficiently. CeO₂ NPs not only promote bone regeneration but also exhibit a long cycle life *in vivo*, further enhancing their therapeutic potential (Dong et al., 2015). The regenerative properties of CeO_2_ NPs are due to the ability of reversible conversion between the crystal structures Ce(III) and Ce(IV) of Ce (Skorodumova et al., 2002), which gives them an enzyme-like activity that is similar to that of *in vivo* SOD, CA, etc., with similar effects (Zou et al., 2018; Hirst et al., 2009). In addition, CeO₂ NPs enhance the proliferation and promote both osteogenic and adipogenic differentiation of BMSCs and MC3T3 preosteoblasts. This dual functionality highlights their potential for applications in bone tissue engineering and regenerative medicine (Wei et al., 2021). Zhang et al. (2023). A multifunctional CeO₂ nanoparticle-reinforced bioactive glass scaffold (CeO₂-BG scaffold) was designed using 3D printing technology. The incorporation of CeO₂ NPs into the BG matrix significantly improved the mechanical properties of the composite while imparting excellent anti-inflammatory properties, high bioactivity, and robust osteogenic potential. The use of 3D printing enabled the scaffold to achieve a porous structure, enhancing its biocompatibility with the implanted bone and ensuring good degradability. In the early stages of implantation, the CeO₂-BG scaffold exhibited strong antioxidant activity, effectively reducing oxidative stress in bone tissue. Additionally, it promoted osteoblast differentiation and bone regeneration by enhancing bone mineralization, increasing ALP activity, and facilitating other regenerative processes (Figure 6).

### 3.6 3D-printed biomaterials with immunomodulatory effects

Usually, when implanting 3D printed scaffolds, M1 macrophages first move to infiltrate the wound area to remove fine debris, and then the transformed M2 macrophages induce angiogenesis and promote tissue repair (Vasconcelos et al., 2019). However, studies have demonstrated that M1 macrophages adhering to biomaterials can maintain or even amplify the production of pro-inflammatory cytokines, including IL-1β, TNF-α, and IL-6, without undergoing a phenotypic shift (Jones et al., 2007), prolonged and elevated cytokine production has been linked to exacerbated tissue injury. Consequently, 3D-printed scaffolds may inadvertently sustain the presence of M1 macrophages, potentially triggering a foreign body reaction (FBR) and resulting in fibrous encapsulation, which could compromise the scaffold’s integration and function. Therefore whether it is related to the reduction of the inflammatory response of the bone tissue surrounding osteoporosis or the reduction of the immune response brought about by the implant itself, the function of biomaterials to regulate macrophage polarization is a key strategy to coordinate the promotion of bone tissue regeneration as well as to improve the biocompatibility of the material further and minimize the negative effects of the material as a human foreign body so as to maximize the promotion of osteoporosis in its own sexual bone regeneration.

#### 3.6.1 Surface modification and mechanical properties of anti-inflammatory 3D printing materials

Immune responses are currently being managed through strategies such as altering the physical properties of materials, incorporating anti-inflammatory agents, or embedding biologics into 3D-printed scaffolds. For instance, niobium-doped 3D-printed porous titanium scaffolds have demonstrated the ability to modulate macrophage polarization, promoting a transition toward a pro-healing phenotype and reducing inflammatory responses. These approaches aim to enhance scaffold biocompatibility and improve regenerative outcomes (Liang et al., 2020/09). Some materials such as chitosan, hyaluronic acid, polylactic acid-glycolide, and other natural or synthetic polymers have natural anti-inflammatory properties that reduce inflammatory responses in the body. Chitosan exhibits the ability to suppress the release of pro-inflammatory cytokines while promoting the production of anti-inflammatory cytokines, making it highly suitable for applications in bone tissue regeneration. Additionally, extracellular matrix (ECM) derived from mesenchymal stem cells (MSCs) isolated from the human endometrium has been shown to effectively modulate anti-inflammatory responses, further supporting its potential in regenerative medicine (Paul et al., 2019). However, the use of derived ECM may lead to pathogen transmission and is more complex to prepare. The loading of drugs on materials has been widely studied, such as antibiotics (Visscher et al., 2018), corticosteroids (Bloomquist et al., 2018) and NSAIDs (Holländer et al., 2016) to drive M2 polarization to suppress immune responses (Alvarez-Lorenzo et al., 2017). For example, researchers incorporated interferon-gamma (IFN-γ) into CaSiO₃-β-TCP 3D-printed scaffolds to achieve dual modulation of macrophage polarization. This approach leverages the synergistic effects of IFN-γ and the controlled release of silicon ions, promoting a balanced immune response conducive to tissue regeneration (Li T. et al., 2018). However, maintaining stable drug or cytokine concentrations in body fluids over extended periods is challenging, and controlling and predicting drug release rates across different sites and bodily fluid conditions remains difficult (Vishwakarma et al., 2016). As a result, modulating macrophage phenotypes using 3D printed scaffolds continues to present a significant challenge.

Liu X et al. developed a novel 3D-printed scaffold by integrating poly (L-lactide) (PLLA) electrospun microfibers (3D-M-EF) and nanofibers (3D-N-EF) into PCL scaffolds through a combination of 3D printing and electrostatic spinning techniques. PLLA fibers were designed to regulate macrophage polarization at the microscopic level, thereby influencing osteogenesis, while the 3D PCL scaffolds provided a macro-scale framework for bone reconstruction. This dual-scale approach resulted in an immunopolarization-modulated 3D-printed bone regeneration scaffold.

Initially, micro- and nanoscale electrospun fibers (M-EF, N-EF) were fabricated using electrostatic spinning technology. The 3D-printed EF scaffolds were then constructed by combining layer-by-layer printing and electrostatic spinning. The 3D-M-EF scaffolds successfully polarized macrophages toward the M2 phenotype, enhanced integrin β1 expression, and activated the PI3K/AKT signaling pathway. These effects facilitated osteoblast differentiation and angiogenesis, leading to the effective repair of bone defects *in vivo*. This study highlights the potential of immunopolarization-modulated scaffolds as a promising strategy for bone regeneration (Garg et al., 2013).

#### 3.6.2 Immune-modulating nanoscale 3D printed materials

Carbon monoxide (CO) is a distinctive endogenous signaling molecule that plays a vital role in regulating inflammation by facilitating the reprogramming of macrophages toward the M2 phenotype. This reprogramming reduces inflammation and promotes tissue repair and regeneration, positioning CO as a key mediator in immune modulation and tissue healing processes (Liu et al., 2020). CO-releasing molecules (CORMs) can solve the problems of difficulty in controlling the dosage and complexity of the way of use of CO as a gas. Among them, hollow manganese dioxide (MnO_2_) nanoparticles have high drug-carrying capacity and biodegradability (Fan et al., 2016). MnO_2_ nanocarriers can safely and efficiently deliver these drugs, avoiding the rapid diffusion of the material in the body after placement; secondly, MnO_2_ has catalase (CAT) activity (Li W. et al., 2017), which can effectively decompose endogenous H_2_O_2_ and release oxygen thereby promoting the intracellular release of CO. Manganese carbonate (MnCO) is an effective precursor drug for carbon monoxide (CO) that releases both CO gas and Mn^2^⁺ upon stimulation by the inflammatory microenvironment, which is characterized by high levels of ROS. This release triggers the polarization of macrophages to the M2 phenotype, significantly reducing the inflammatory response and promoting tissue healing (Zhang et al., 2020). Desferrioxamine (DFO), an iron chelator, plays a significant role in enhancing vascularization and facilitating the regeneration of vascularized bone. Its mechanism involves upregulating hypoxia-inducible factor 1α (HIF-1α), a critical regulator of cellular responses to hypoxic conditions. This promotes angiogenesis, accelerates the formation of new blood vessels, and enhances bone healing, making DFO a promising therapeutic agent in bone regeneration (Duscher et al., 2019). In addition, Desferrioxamine (DFO) inhibits osteoclast differentiation by disrupting the electron transport chain and downregulating the activation of the mitogen-activated protein kinase (MAPK) pathway (Zhang et al., 2019b). However, its high water solubility causes rapid local release and excessive accumulation, which can lead to significant tissue toxicity. This limitation highlights the need for strategies to control its release and mitigate adverse effects while preserving its therapeutic benefits (Han et al., 2021). Zhang J et al. developed a 3D-printed hybrid scaffold composed of Desferrioxamine (DFO) and manganese carbonate (MnCO) incorporated into a gelatin methacryloyl-polylactide (DMGP) matrix. The scaffold featured a biomimetic porous structure, achieved by immersing alkali-treated poly (lactic acid) (PLA) in a hydroxyapatite (HA)/ethanol suspension. This innovative design was tailored to enhance bone regeneration and vascularization through the controlled release of DFO and MnCO, leveraging their synergistic effects on osteogenesis and angiogenesis (Zhang et al., 2022). The sustained release of CO and Mn^2+^ by MnCO in the presence of H_2_O_2_ at the site of inflammation upregulated the M2 polarization phenotype of macrophages thereby significantly ameliorating the inflammatory response, and the scaffolds enabled the controlled release of DFO to exert the immune-modulatory osteogenesis and angiogenesis-promoting ability of DFO at defined local drug concentrations. Angiogenesis is further enhanced by Mn^2^⁺ activation of the HIF-1α pathway, which stimulates the secretion of VEGF from M2 macrophages. In *in vivo* and *in vitro* studies, this scaffold showed good bone immunomodulatory properties such as reducing inflammation, promoting angiogenesis, inhibiting osteoclastogenesis, and better osteogenic capacity, which had a significant effect on improving bone regeneration.

3D-printed porous titanium (PT) has good osseointegration properties and stimulates bone regeneration though (Zadpoor, 2019; Charbonnier et al., 2021). However, when PT is used as an *in vivo* bone implant, the bonding with bone tissue only occurs near the interface with bone (Pobloth et al., 2018), so in the case of osteoporosis, the decrease in bone density will greatly reduce the contact area between the material and bone tissue, which significantly diminishes the osteogenic properties of PT. Research has shown that localized drug delivery systems can directly impact the microenvironment at the implant site, providing an effective means of modulating bone metabolism. This approach has gained considerable attention in the field of regenerative medicine. Recent progress in nanomaterials has further expanded the potential for developing controlled-release drug delivery systems, enabling more precise and sustained therapeutic effects to enhance bone healing and regeneration (Mohammadi et al., 2018; Li Z. et al., 2021). MOFs have attracted significant interest for their potential to promote osteogenesis, attributed to their customizable composition, advantageous chemical properties, extensive surface area, biodegradability, and high porosity. Hu et al. investigated the drug delivery capabilities of MOF-74, highlighting its low cytotoxicity and controlled drug release properties. These findings confirm that MOFs are excellent carriers for drug delivery, positioning them as promising candidates for applications in bone tissue engineering and related therapeutic strategies (Hu et al., 2014). Shen et al. developed Mg/Zn-MOF coatings for titanium surfaces, demonstrating that the controlled release of Zn^2^⁺ and Mg^2^⁺ ions during the biodegradation of the coatings could effectively stimulate new bone formation while providing antibacterial properties. These dual functions highlight the potential of Mg/Zn-MOF coatings in enhancing the performance of titanium implants for bone tissue engineering applications (Shen et al., 2019). However, MOFs exhibit instability in acidic environments, which poses a challenge since the microenvironment of osteoporosis is typically acidic. Therefore, further modification of MOFs is necessary for their effective use in orthopedic applications. Additionally, Mg^2^⁺ ions have been found to play a significant role in bone tissue regeneration by modulating the activity of osteoblasts and macrophages, making them a promising component for enhancing bone repair processes (O'Neill et al., 2018). Wang W et al. developed a novel biofunctionalized 3D-printed porous Ti6Al4V scaffolds with macroscopic/micro/nanoscopic scales loaded with epimedium glycosides to obtain ICA@MOF (Wang et al., 2022), which was investigated using a PT/filiprotein (SF) composite scaffold. SF can form an extracellular matrix (ECM)-like structure within PTs through freeze-drying, creating a favorable microenvironment for cell adhesion and proliferation. The integration of MOFs into PTs, encapsulated by SF, enhances the stability of the MOFs. The biodegradable SF network not only provides mechanical support for cell attachment but also releases degradation products that serve as nutrients for cell growth, simultaneously promoting bone tissue regeneration.

**FIGURE 7:**
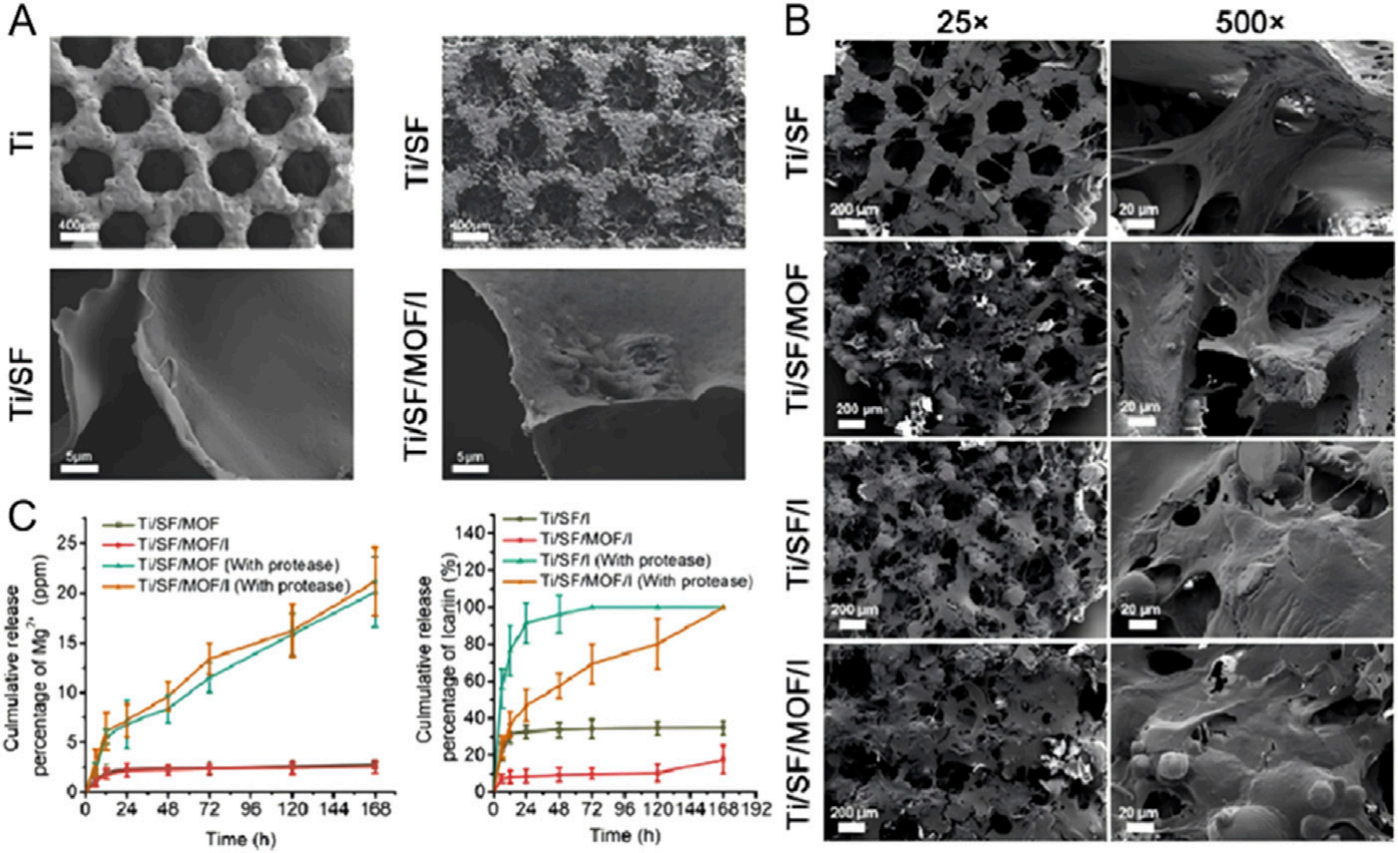
Icebergamycin was successfully loaded onto hierarchically biofunctionalized 3D-printed porous Ti6Al4V scaffolds (PT) (Wang et al., 2022). The scaffold’s porous architecture not only provides mechanical support but also enhances cell adhesion and biocompatibility. These PT scaffolds enable controlled release of icariin and Mg^2^⁺ ions, effectively promoting the polarization of M0 macrophages toward the M2 phenotype by inhibiting the Notch1 signaling pathway. This macrophage polarization leads to the secretion of anti-inflammatory cytokines, significantly improving bone metabolism. At the microscale, a biomimetic extracellular matrix (ECM) was established within the 3D-printed porous Ti6Al4V scaffolds, mimicking natural ECM for enhanced cellular interaction. At the nanoscale, icariin@Mg-MOF-74 (ICA@MOF) was encapsulated within the ECM-like structure, ensuring controlled release of icariin and Mg^2^⁺ ions. This hierarchical integration allows for precise modulation of the local biochemical environment, promoting bone regeneration. **(A)** Micromorphology of the samples, as visualized through scanning electron microscopy (SEM). **(B)** Adhesion morphology of BMSCs on the scaffold surfaces. **(C)** Release profiles of icariin and Mg^2^⁺ from various biofunctionalized PT scaffolds over time in protease-free and protease-containing PBS solutions. This advanced scaffold design highlights the potential of combining hierarchical structural features with controlled drug delivery to enhance bone tissue regeneration. [Reproduced with Open Access from Wang, W., Xiong, Y., Zhao, R. et al. A novel hierarchical biofunctionalized 3D-printed porous Ti6Al4V scaffold with enhanced osteoporotic osseointegration through osteoimmunomodulation. *J Nanobiotechnol* 20, 68 (2022)].

In this hierarchically biofunctionalized porous Titanium (PT) implant, the macroscopic large pore size of the PT material offers critical mechanical support, while the ECM-like SF structure facilitates cellular activity in the microscopic environment. At the nanoscale, the incorporated MOFs contribute biological effects, such as promoting the polarization of M0 macrophages to the M2 phenotype. This polarization enables the sustained release of epimedium glycosides and Mg^2^⁺ ions, which stimulate the secretion of anti-inflammatory factors. These actions modulate bone metabolism via immune pathways, significantly improving the osteoporotic environment and enhancing osseointegration. This multi-scale approach underscores the scaffold’s potential for advanced bone regeneration applications (Figure 7).

#### 3.6.3 Immunomodulating bio-glass and bio-ceramic 3D printing materials

**FIGURE 8:**
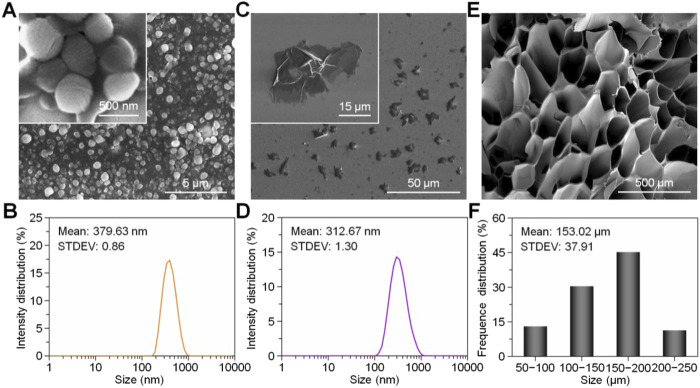
Physical properties of composite hydrogels. Scanning electron microscopy images of **(A)** DFO@PCL nanoparticles, **(C)** MnCO nanosheets, and **(E)** GelMA hydrogel. **(B)** Particle size distribution of DFO@PCL nanoparticles and **(D)** MnCO nanosheets. **(F)** Pore size distribution of GelMA hydrogel. Figure 8 is reproduced from Ref Zhang et al. (2022) with permission from Wiley-VCH GmbH.

In addition, silicon-based materials play a pivotal role in enhancing osteoblast activity, promoting bone mineralization, and supporting normal bone metabolism. Studies have demonstrated that optimal concentrations of silicon can significantly stimulate the proliferation of MC3T3-E1 cells by enhancing the production of bone-specific proteins (Shie et al., 2011). Furthermore, silicon contributes to neovascularization, with silicon-containing bioglasses directly or indirectly promoting the secretion of VEGF, thereby facilitating angiogenesis and supporting bone tissue regeneration (Zhai et al., 2012). Deng Y et al. demonstrated that 5% CaSiO₃-β-TCP scaffolds exhibited superior angiogenic and osteoinductive capabilities compared to β-TCP alone. These scaffolds effectively stimulated the secretion of platelet-derived growth factor-BB (PDGF-BB) and stromal cell-derived factor 1 (CXCL12) into the surrounding environment, thereby enhancing tissue regeneration and vascularization (Deng et al., 2017). Additionally, silicate has been shown to polarize macrophages towards a pro-fibrotic M2 phenotype through the macrophage receptor with collagen structure (MARCO) (Murthy et al., 2015). Silicates not only directly stimulate angiogenesis, as previously noted, but may also indirectly promote macrophage polarization toward the M2 phenotype. This polarization facilitates tissue remodeling and supports the development of mature neovascularization. Li T et al. developed a 3D-printed CaSiO₃-β-TCP scaffold loaded with IFN-γ through physical adsorption. This innovative scaffold design combines the angiogenic and macrophage-modulating properties of silicates with the immunoregulatory effects of IFN-γ, enhancing its potential for vascularized bone regeneration (Li T. et al., 2018). The scaffold initially induced M1 macrophage polarization to enhance chemotaxis, guiding macrophages to the inflammation site for pathogen clearance. Research has shown that TNF-α and IL-1β, secreted by M1 macrophages, play a critical role in activating tip cells, stimulating vascular endothelial cell proliferation, and recruiting perivascular cells. These processes collectively establish a favorable environment for angiogenesis during the later stages of tissue repair and regeneration (Sainson et al., 2008; Wynn et al., 2013). Following the initial M1 polarization, subsequent stimulation of M2 macrophage polarization further recruits perivascular cells and mesenchymal stem cells, aiding in the remodeling of early vascular sprouts and enhancing their stability and maturation (Ponte et al., 2007; Stratman et al., 2010). Specifically, M2a-type macrophages secrete tissue inhibitors of matrix metalloproteinase-3 (TIMP3), which inhibits MMP9 activity and prevents its binding to VEGFR2. This action suppresses VEGF signaling, thereby regulating angiogenesis. Moreover, TIMP3 also inhibits the release of TNF-α, enabling late-stage M2 macrophages to not only modulate angiogenesis but also influence signaling pathways in M1 macrophages, ensuring a coordinated and balanced immune response during tissue repair and regeneration (Figure 8).

#### 3.6.4 Immune-modulating metal 3D printing materials

Cerium, as a metallic rare earth element, has excellent catalytic activity. Cerium in crystalline CeO_2_ exists in a mixed valence state of Ce^3+^ and Ce^4+^ and can be used as a catalytic site on the surface of materials (Thakur et al., 2019; Malyukin et al., 2017). Cerium oxide nanoparticles (CeONP) have been shown to undergo an enzymatic-like catalytic reaction with several ROS, with larger values of Ce^3+^/Ce^4+^ being more SOD-active, and conversely, smaller values of Ce^3+^/Ce^4+^ being more peroxidase-active (Pirmohamed et al., 2010), which further affects macrophage polarization. Studies have reported that CeONPs scaffolds promote BMSC adhesion and proliferation (Xiang et al., 2016). And CeONPs simultaneously reduced the release of pro-inflammatory mediators IL-6, IL-1β, inducible nitric oxide synthase (iNOS), and TNF-α from mouse macrophages (Hirst et al., 2009; Selvaraj et al., 2015). Wei F et al. studied the effects of varying concentrations of CeONPs *in vitro* under both acute and chronic inflammatory conditions, focusing on their ability to modulate macrophage phenotypes and regulate the release of inflammatory cytokines. The findings revealed that CeONPs consistently suppressed iNOS expression during the early phase of inflammation. Furthermore, at lower doses, CeONPs significantly reduced the expression of pro-inflammatory cytokines IL-1 and IL-6, demonstrating their potential as effective anti-inflammatory agents (Wei et al., 2021).

Magnesium can play an important immunomodulatory role in bone tissue regeneration. Mg^2^⁺ regulate immune responses via TRPM7 and M7CKs channels, inducing macrophages to secrete anti-inflammatory and osteogenic factors. Additionally, Mg^2^⁺ activates key signaling pathways, including the MAPK/ERK and Wnt/β-catenin pathways. These pathways play a critical role in promoting the proliferation of BMSCs and enhancing their osteogenic differentiation, making Mg^2^⁺ a vital element in bone regeneration and immune modulation (Zhou et al., 2021/05; Wang et al., 2022/12; Qiao et al., 2021). The addition of magnesium to bone repair materials such as titanium alloy and tricalcium phosphate has been demonstrated in previous studies to improve their osteogenic properties (Wei et al., 2010; Hou et al., 2023/01). Dai K et al. prepared a micro-nano bioactive glass (MNBG) with good bioactivity (Dai et al., 2024), homogeneous size distribution, and regular morphology using PLGA and PCL as carriers using 3D printing technology. And magnesium was added to MNBG, which can release Mg^2+^ and Si^4+^ and Ca^2+^ simultaneously (Zeimaran et al., 2015). The MNBG can induce mineralization to promote bone tissue regeneration by releasing Si^4+^ and Ca^2+^. Moreover, the scaffold has a more stable degradation rate, which can avoid excessive Mg^2+^ concentration in the early implantation period and thus inhibit bone regeneration by stimulating the NF-κB signaling pathway.

## 4 Conclusion and prospects

Bone defects in osteoporosis present a more complex microenvironment and mechanism of action compared to those in normal bone, characterized by prolonged high bone turnover rates and reduced osteogenic activity. Standard osteoporosis treatments are often inadequate for achieving high local drug concentrations necessary to promote bone regeneration in defect sites effectively. As a result, current approaches often rely primarily on suppressing osteoclast activity, which limits the efficacy of bone repair.

In osteoporotic bone defects, the pathological microenvironment is dominated by chronic inflammation. ROS exacerbate this inflammatory milieu, promoting the polarization of macrophages toward the M1 phenotype, which further impairs bone healing. Therefore, the development of bone-implantable biomaterials with integrated osteogenic, anti-inflammatory, and antioxidant properties is crucial for addressing the challenges associated with bone defects in osteoporosis. These multifunctional materials have the potential to modulate the local inflammatory environment, reduce oxidative stress, and enhance bone regeneration, offering a promising strategy for effective bone repair in osteoporotic conditions.

Autologous bone promotes bone formation on the bone surface through direct bone integration and induces BMSCs to differentiate into osteocytes. Therefore, it is still considered the most effective method of bone regeneration in the clinic without any associated immune response (Zhang et al., 2014). However, the limited supply of autologous bone and the damage to the donor site are not negligible disadvantages (Goulet et al., 1997). Allogeneic bone, on the other hand, while having similar functions, greatly increases the risk of allograft rejection at the defect site and infection by other pathogens that are difficult to predict (Hill and Purtill, 2022).

3D printed implantable biomaterials have significant advantages today because they can be manufactured according to different structures and sites to match their space-sustaining materials, first of all, the mechanical properties of the material itself, the release of substances, the role of support, etc., will have different anti-osteoporosis effect, so past research has developed a variety of such as metal, bioceramics, bio-active glass, composites, etc., and the material is processed differently to make it have different macroscopic and microscopic properties. They have different macroscopic and microscopic properties, for example, the porous structure has similar mechanical properties to cancellous bone, the calcium-phosphorus ceramic material can bind with BMP2 to promote osteogenesis, the release of metal ions Mg^2+^ and Zn^2+^ has an anti-inflammatory effect, and the rare-earth material can effectively neutralize the accumulation of ROS. The material piggybacking drug can maintain high and stable drug concentration locally, sustaining the inhibitory effect on local inflammation and ROS, and reducing systemic side effects.

3D-printed biomaterials can be customized to meet the specific needs of patients. Some titanium implants made using 3D printing technology are already in clinical use. By adjusting the material’s porous structure, the mechanical properties can be optimized to match the patient’s bone tissue. In the treatment of severe osteoporotic vertebral compression fractures (SOVCF), 3D printing digital technology has been used to assist in modifying crossed percutaneous vertebroplasty (PVP). This approach reduced operative time and puncture positioning time, decreased fluoroscopy, significantly reduced patient pain, improved lumbar spine function and spino-pelvic sagittal balance, improved quality of life, and reduced the risk of cement leakage. Ongoing studies have shown that the combination of mesenchymal stem cells (MSCs) with 3D printed scaffolds exhibits potential in osteoporosis treatment (He et al., 2021). 3D printed scaffolds provide a favorable microenvironment for stem cells to grow and promote their differentiation towards osteoblasts to repair bone defects caused by osteoporosis. In addition, exosome-driven drug delivery systems are also receiving attention in this field.

Despite the significant development, the challenge still exists that the implantation of materials is not only a localized system. 3D printed biomaterials should not be limited to the treatment of osteoporosis in the local microenvironment, but also have a good synergistic effect with the whole body’s physiological response, and explore the mechanism of action of the materials and the drugs they carry over the entire body. Barriers to the translation of 3D-printed biomaterials, in addition to the different drawbacks of each of the different materials outlined in the article, include low throughput due to inefficiencies in 3D-printing technology; insufficient clinical data currently available and a lack of clinical data for long-term follow-up; the high cost of certain materials and the tension between material customization and implant standards. In addition to the influence of the human body environment on the material, clinical safety and clinical feasibility also determine whether the biomaterials can eventually be converted to clinical use, the high degree of matching of 3D printing technology will also bring the issue of whether the material can be mass produced, so in 3D printing biomaterials to really move towards clinical research requires the joint efforts of experts from various disciplines.
